# Whole-Genome Analysis of Temporal Gene Expression during Foregut Development

**DOI:** 10.1371/journal.pbio.0020352

**Published:** 2004-10-19

**Authors:** Jeb Gaudet, Srikanth Muttumu, Michael Horner, Susan E Mango

**Affiliations:** **1**Huntsman Cancer Institute, University of UtahSalt Lake City, UtahUnited States of America

## Abstract

We have investigated the *cis*-regulatory network that mediates temporal gene expression during organogenesis. Previous studies demonstrated that the organ selector gene *pha-4*/FoxA is critical to establish the onset of transcription of Caenorhabditis elegans foregut (pharynx) genes. Here, we discover additional *cis*-regulatory elements that function in combination with PHA-4. We use a computational approach to identify candidate *cis*-regulatory sites for genes activated either early or late during pharyngeal development. Analysis of natural or synthetic promoters reveals that six of these sites function in vivo. The newly discovered temporal elements, together with predicted PHA-4 sites, account for the onset of expression of roughly half of the pharyngeal genes examined. Moreover, combinations of temporal elements and PHA-4 sites can be used in genome-wide searches to predict pharyngeal genes, with more than 85% accuracy for their onset of expression. These findings suggest a regulatory code for temporal gene expression during foregut development and provide a means to predict gene expression patterns based solely on genomic sequence.

## Abstract 2

Computational analysis combined with validation by reporter gene studies is uncovering the code for temporal gene regulation in the *C. elegans* foregut - a model for organogenesis

## Introduction

Formation of organs depends on successive programs of gene expression during development. Temporal regulation of transcription is critical to achieve spatial patterning, as seen during vertebrate somitogenesis (Pourquie 2003). Temporal regulation is also essential to integrate the progression of events that accompany cell fate specification and differentiation (Bruhn and Cepko 1996; Isshiki et al. 2001; Pearson and Doe 2003). The regulatory networks that guide these processes depend on a wide array of transcription factors, raising the question of how transcriptional circuitry dictates developmental timing. In some cases, tiers of transcription factors function hierarchically to establish sequential patterns of gene expression (Kornberg and Tabata 1993; Maduro and Rothman 2002; Skeath and Thor 2003). These regulators are active for only a brief time during development and typically produce a uniform response in expressing cells. For example, three consecutive waves of GATA transcription factors establish the Caenorhabditis elegans midgut (Maduro and Rothman 2002). Ectopic expression of at least some of these GATA factors can convert the entire embryo into midgut, suggesting a homogeneous and robust transcriptional response by these cells. In contrast, other transcriptional regulators function continuously during development (Weatherbee et al. 1998; Bergstrom et al. 2002; Gaudet and Mango 2002). These proteins activate different target genes at different developmental stages, suggesting a more complex, heterogeneous transcriptional response. A critical question is how the second class of transcriptional regulators establishes consecutive programs of gene expression.

The forkhead box (Fox) A family of transcription factors illustrates the second strategy of developmental control. These proteins are critical to form the digestive tract in *Drosophila,* mammals, and worms, and animals lacking FoxA have profound gut defects (Weigel et al. 1989; Ang and Rossant 1994; Mango et al. 1994; Weinstein et al. 1994; Dufort et al. 1998). For example, inactivation of *C. elegans pha-4* leads to a loss of foregut cells, which are transformed into ectodermal cell types such as glia and epidermis (Mango et al. 1994; Horner et al. 1998). This dramatic phenotype reflects the global requirement for PHA-4 to transcribe genes selectively expressed in foregut cells throughout development. Direct PHA-4 targets include early-acting developmental regulators, such as *ceh-22*/Nkx 2–5, and terminal differentiation genes that encode structural proteins and digestive enzymes (Kalb et al. 1998; Gaudet and Mango 2002; Vilimas et al. 2004). FoxA members in other organisms have a similar range of early- and late-expressed targets, suggesting they, too, function during multiple stages of development (Gualdi et al. 1996; Lehmann and Korge 1996; Duncan et al. 1998; Roth et al. 1999; Lee et al. 2002).

The diversity of FoxA target genes raises the question of how these factors achieve appropriate temporal regulation of transcription during development. One answer is affinity for DNA. C. elegans PHA-4 recognizes sequences that conform to the consensus TRTTKRY (where R = A/G, K = T/G, and Y = T/C) (Overdier et al. 1994; Gaudet and Mango 2002). Sequences that bind PHA-4 with high affinity in vitro are typically found in promoters of genes expressed early in development, whereas low-affinity sites are restricted to late promoters (Gaudet and Mango 2002). Moreover, adjustment of a high-affinity binding site to a lower one shifts the onset of expression later, and, conversely, mutation to a higher-affinity site leads to earlier activation (Gaudet and Mango 2002). These data demonstrate that binding-site affinity of PHA-4 for DNA is a critical determinant of gene expression. However, the affinity of PHA-4 for its recognition sequence is not an absolute predictor of gene activation. For example, the pharyngeal muscle myosin gene, *myo-2,* possesses high-affinity PHA-4 sites but is activated late in development (Okkema et al. 1993; Gaudet and Mango 2002). These observations suggest additional factors function in combination with PHA-4 for temporal control of pharyngeal gene expression.

In this study, we have combined bioinformatics and experimental approaches to investigate the *cis*-regulatory network for temporal gene expression within the pharynx. We identify sites that function in combination with PHA-4 elements to distinguish early from late expression. These elements can be used to build synthetic promoters with the expected expression profiles and to identify previously undiscovered pharyngeal genes within the genome.

## Results

**Figure 1:**
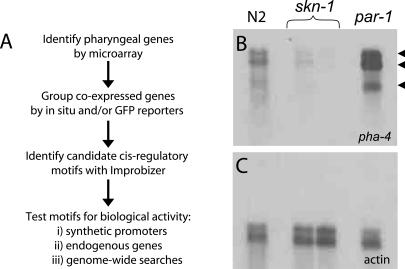
Strategy to Identify Temporal Regulatory Elements (A) Flowchart of the strategy used. (B) Northern blot of *pha-4. pha-4* transcripts were approximately 25- to 100-fold enriched in *par-1* compared to *skn-1* embryos, but only approximately 5- to 10-fold enriched in wild-type compared to *skn-1* embryos. Arrowheads indicate the three different *pha-4* isoforms. (C) The same blot was probed with a fragment of the *act-1* gene to demonstrate equal loading of RNA between lanes.

Our goal was to discover new regulators of pharyngeal transcription that would function in combination with PHA-4 (Figure 1A). To achieve this aim, we first identified candidate pharyngeal genes by microarray analysis and subdivided these genes into clusters based on their onset of expression (early versus late). Next, we searched for short sequences enriched within the predicted promoters of genes from the early or late clusters. These sequences were tested for enhancer or repressor activity in vivo using both natural and synthetic promoters.

### Identification of Pharyngeal Genes

We previously discovered pharyngeal genes using microarrays encompassing 62% of C. elegans genes (Gaudet and Mango 2002). We extended this analysis by screening microarrays that covered 94% of C. elegans genes (17, 871 genes; Jiang et al. 2001). To maximize the sensitivity of detection, we compared gene expression profiles from embryos with excess pharyngeal cells (*par-1;* Kemphues et al. 1988) to embryos with no pharyngeal cells (*skn-1;* Bowerman et al. 1992). *par-1* mutants affect the earliest embryonic cell divisions and produce cell fate transformations, such that *par-1* mutant embryos lack gut cells, but have excess pharynx and body wall muscles. In contrast, *skn-1* mutants lack both gut and pharynx, but have excess body wall muscle and epidermis. Thus, genes with a relatively high *par-1/skn-1* ratio were likely to be selectively expressed in the pharynx. An advantage to using *par-1* and *skn-1* mutants was that they provided a broad range of expression differences. For example, *pha-4* transcripts were approximately 25- to 100-fold enriched in *par-1* versus *skn-1* embryos, compared to only approximately 5- to 10-fold enriched in wild-type versus *skn-1* embryos (Figure 1B and 1C).

We identified 339 genes with at least 2-fold greater expression in *par-1* embryos compared to *skn-1* mutants (Materials and Methods; Table S1). For genes whose expression was known, 81% (114/141) were selectively expressed in the pharynx (Table S1). Importantly, the sensitivity of this approach enabled us to detect genes expressed at low levels (e.g., *ceh-22;* Okkema and Fire 1994; Vilimas et al. 2004) or in a small subset of pharyngeal cells (e.g., C49G7.4; Ao et al. 2004).

### Temporal Groups of Pharyngeal Genes

We categorized the pharyngeal genes into early versus late temporal groups using previously defined expression patterns. The Nematode Expression Pattern Database (Kohara 2001; http://nematode.lab.nig.ac.jp/db/index.html) and green fluorescent protein (GFP) reporters enabled us to identify 37 early-onset and 34 late-onset pharyngeal genes (Table S2). The set of early-expressed pharyngeal genes (Ph-E) contained genes whose expression initiated by mid-embryogenesis (the “bean” to “comma” stages; Sulston et al. 1983). At the end of this developmental stage, embryonic cell division is virtually complete, the pharynx primordium has formed, and cell fate patterning of the primordium has begun. The set of late-expressed pharyngeal genes (Ph-L) contained genes activated at the onset of terminal differentiation of the pharynx (the 3-fold stage; Sulston et al. 1983). Forty-three genes were associated with expression patterns but were not assigned to either category either because the onset of expression was ambiguous or because it fell between the early and late categories.

**Figure 2:**
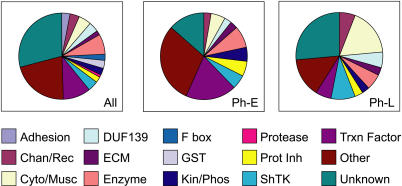
Different Predicted Products of the Temporal Groups The Ph-E group is enriched for predicted transcription factors, while the Ph-L group is enriched for predicted structural or muscle proteins (see Table 1). Muscle proteins are proteins known or predicted to be involved in muscle function, including myosins, tropomyosins, and troponins. Table S2 provides a complete listing of the catergorization of the Ph-E and Ph-L genes.

The Ph-E cluster was enriched for genes predicted to encode transcription factors (Figure 2; Table 1), consistent with Ph-E genes controlling early aspects of pharyngeal development such as cell fate specification. The Ph-L group was enriched for genes predicted to encode cytoskeletal or muscle proteins (Figure 2; Table 1), consistent with Ph-L genes being involved in terminal differentiation and pharyngeal function. Intriguingly, Ph-L genes were more likely to be located on Chromosomes V and X (*p* ≤ 0.05) at the expense of Chromosomes I and IV (*p* ≤ 0.06; Table 2). Biases for gene placement on chromosomes have been observed previously. For example, genes expressed in the male germ line are excluded from the X chromosome (Reinke et al. 2000), while muscle genes are often clustered along a chromosome (Roy et al. 2002).

**Table 1:**
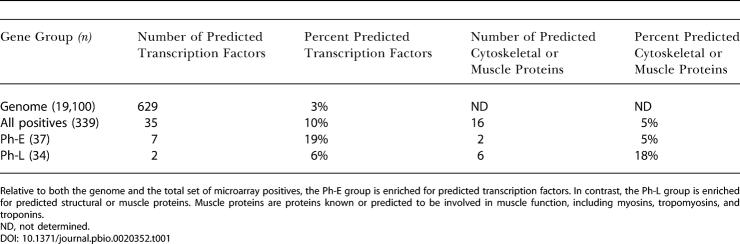
Temporal Groups of Pharyngeal Genes Are Enriched for Different Kinds of Predicted Products

Relative to both the genome and the total set of microarray positives, the Ph-E group is enriched for predicted transcription factors. In contrast, the Ph-L group is enriched for predicted structural or muscle proteins. Muscle proteins are proteins known or predicted to be involved in muscle function, including myosins, tropomyosins, and troponins

ND, not determined

**Table 2:**
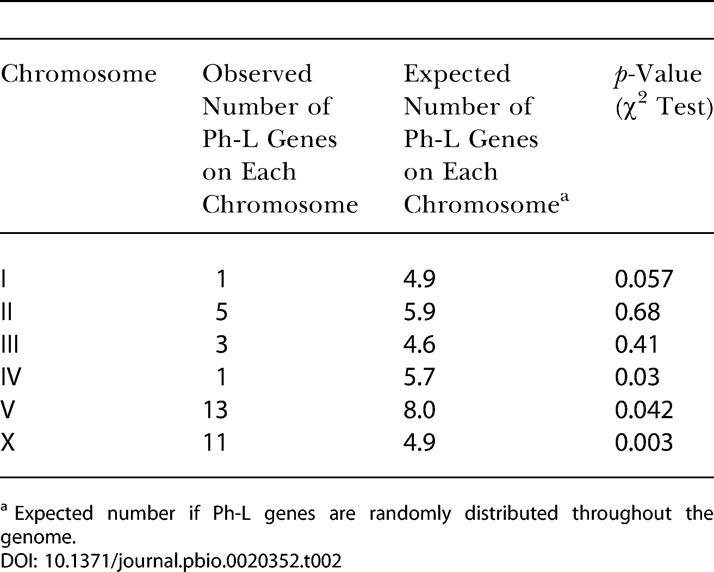
Ph-L Genes Are Enriched on Chromosomes V and X

^a^ Expected number if Ph-L genes are randomly distributed throughout the genome

### Identifying Regulatory Elements in Pharyngeal Promoters

We examined predicted promoters of Ph-E and Ph-L members for candidate *cis*-regulatory elements that might contribute to temporal regulation. We first estimated the size of pharyngeal promoters by determining the sequence identity between pairs of C. elegans and C. briggsae orthologs. Conservation is a good indicator of functionally important regions for *cis*-regulation (Kirouac and Sternberg 2003; Hwang and Sternberg 2004; Liu et al. 2004). We scored as “conserved” those regions of DNA with 75% or greater identity over 50 bp. Sixty-six percent of pharyngeal genes had conserved sequences within 500 bp upstream of the predicted start codon (*n =* 64 genes; Table 3), whereas only 21% had conserved sequences from 500 to 1,000 bp (*n =* 63 genes; Table 3). This landscape of sequence conservation agreed well with reporter studies in which 500 bp of upstream sequence was often sufficient to recapitulate the endogenous pattern of expression (Gaudet et al. 1996; McGhee and Krause 1997; Gaudet and Mango 2002). Based on these observations, we chose to limit our motif searches to 500 bp upstream of predicted start codons.

**Table 3:**
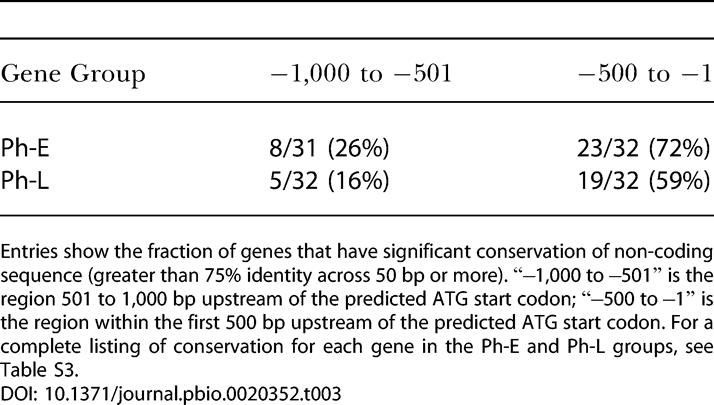
Conservation of Non-Coding Sequence between C. elegans and C. briggsae

Entries show the fraction of genes that have significant conservation of non-coding sequence (greater than 75% identity across 50 bp or more). “−1,000 to −501” is the region 501 to 1,000 bp upstream of the predicted ATG start codon; “−500 to −1” is the region within the first 500 bp upstream of the predicted ATG start codon. For a complete listing of conservation for each gene in the Ph-E and Ph-L groups, see Table S3

**Figure 3:**
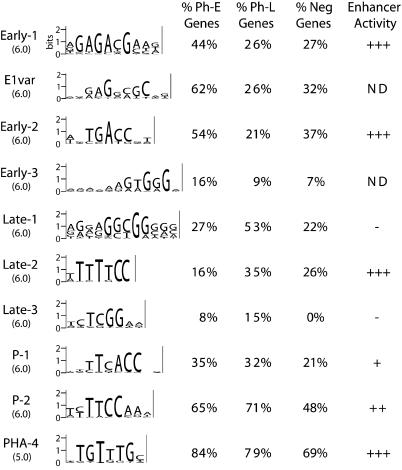
Candidate Pharyngeal Motifs Identified by Improbizer Improbizer represents motifs as PWMs; the PWMs for the motifs are shown in Dataset S1. We converted these matrices to the “sequence logos” shown here (Schneider and Stephens 1990; Crooks et al. 2004). The threshold scores used by Cluster-Buster (Frith et al. 2003) for each motif are shown below the sequence logos. “% Ph-E Genes” and “% Ph-L Genes” are the percentage of Ph-E and Ph-L genes that contain occurrences of a given motif within 500 bp upstream of the predicted ATG start codon, above the threshold shown. “% Neg Genes” is the percentage of DNA synthesis genes that contain occurrences of a given motif (above the threshold score) within 500 bp upstream of the predicted ATG start codon. Two other negative groups (carbohydrate synthesis genes [Kim et al. 2001] and a set of 194 randomly selected genes) yielded similar results (data not shown). “Enhancer Activity” is the relative strength of expression generated by a motif present in three copies upstream of the Δ*pes-10::GFP::HIS2B* reporter. ND, not determined.

We used the Improbizer expectation maximization algorithm to search pharyngeal promoters for potential regulatory elements (Ao et al. 2004). Improbizer detects short sequences that are over-represented within a cohort of genes. As a negative control, we examined groups of genes that were not specifically expressed in the pharynx (e.g., DNA synthesis genes or a set of 194 randomly selected genes) and removed motifs common to both the pharyngeal and negative control sets. This comparison identified nine candidate regulatory motifs for pharyngeal genes (Figures 3; Dataset S1). Significantly, two motifs, Early-3 and P-3, conformed to the consensus sequences for the pharyngeal transcription factors CEH-22 and PHA-4, respectively (Okkema and Fire 1994; Kalb et al. 1998; Gaudet and Mango 2002), indicating that our approach could successfully identify pharyngeal regulatory elements.

Five of the remaining seven motifs were associated with either the Ph-E or Ph-L temporal expression groups (Figure 3). The Early-1 and Early-2 motifs were found by screening promoters of the Ph-E gene group and occurred more frequently in Ph-E promoters (44% and 54% of promoters, respectively) than in Ph-L promoters (26% and 21% of promoters, respectively). Conversely, the Late-1, Late-2, and Late-3 elements were found in Ph-L promoter searches and were more likely to occur in Ph-L promoters (53%, 35%, and 15% of promoters) than in Ph-E promoters (27%, 16%, and 8% of promoters). Two other motifs named P-1 and P-2 (for pharyngeal gene motifs 1 and 2) were discovered by screening Ph-E and Ph-L promoters together. These elements occurred in Ph-E and Ph-L genes with comparable frequency and were more frequently represented in promoters of pharyngeal genes (P-1 in 34% and P-2 in 68% of promoters) than of non-pharyngeal control genes (P-1 in 21% and P-2 in 48% of promoters). These data suggest that P-1 and P-2 are pharyngeal regulatory elements that are not associated with temporal control.

We reexamined Ph-E genes for additional elements present in genes that contained neither Early-1 nor Early-2 elements. Given that 48% of Ph-E genes contained one or more Early-1 or Early-2 elements (Table S4), we examined the remaining 52% of Ph-E genes for the presence of additional motifs. This survey identified a single motif that appeared to be a variant of the Early-1 motif and was therefore named “E1var.” As with Early-1, E1var was enriched in the promoters of Ph-E pharyngeal genes compared to Ph-L pharyngeal and non-pharyngeal genes (Figure 3). No other motifs were identified above background in these follow-up searches. We performed a similar analysis of Ph-L genes that did possessed neither Late-1 nor Late-2 elements, but found no additional motifs. These results suggest that if other temporal elements exist, they are represented by relatively degenerate sequences, are shared by small numbers of genes, occur in both pharyngeal and non-pharyngeal genes, or are outside the 500-bp regions examined.

### In Vivo Activity of Candidate Pharyngeal Regulatory Elements

Two tests demonstrated that six of the candidate motifs had biological activity. First, we performed “enhancer assays” to determine whether a motif was sufficient to activate expression when introduced into a heterologous basal promoter. Second, we used site-directed mutagenesis to inactivate a motif within a native pharyngeal promoter and examine whether it was necessary for expression.

**Figure 4:**
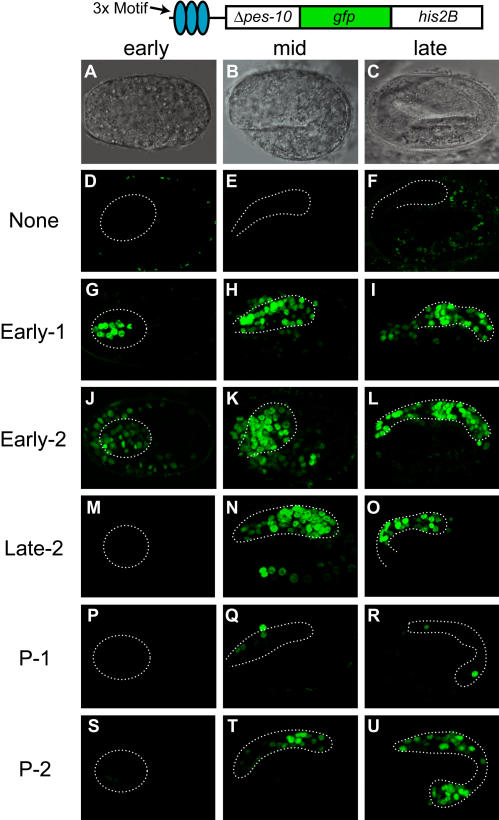
Five Newly Identified Motifs Function as Pharyngeal Enhancers (A–C) Nomarski differential contrast interference images of embryos representing three different stages of embryonic development: (A) “early” development, when the pharynx primordium is formed, (B) “mid” development, when the pharynx has completed cell division and attached to the presumptive buccal cavity, and (C) “late” development, when pharynx development is almost complete and the embryo is about to hatch. Images on the left are of “early” embryos, images in the middle are of “mid” embryos, and images on the right are of “late” embryos. (D–U) Representative transgenic embryos showing expression from reporter constructs containing the Δ*pes-10* promoter alone (D–F) or with insertion of three copies of Early-1 (G–I), Early-2 (J–L), Late-2 (M–O), P-1 (P–R), or P-2 (S–U). Dashed lines indicate the outline of the developing pharynx.

We used the Δ*pes-10* promoter for the heterologous enhancer assays (Figure 4). This promoter does not activate GFP (Figure 4D–4F) but is competent to respond to enhancers in most or all tissues (Seydoux and Fire 1994; Fire et al. 1998). Previous studies established that the PHA-4 binding site and the CEH-22 binding site could activate expression of Δ*pes-10::GFP* in pharyngeal and pharyngeal muscle cells, respectively (Kuchenthal et al. 2001; Vilimas et al. 2004). We therefore used this reporter to determine whether our motifs could function as pharyngeal enhancers.

Our heterologous promoter assay demonstrated that five of the seven pharyngeal motifs could function as enhancers in vivo. Early-1 and Early-2 activated pharyngeal expression early, after specification of pharyngeal precursors at the 200-cell stage but before formation of the pharynx primordium (Figure 4G–4L). In both cases, activity was confined to embryos. Early-1 was active in most or all pharyngeal cells, with occasional activity in non-pharyngeal cells. Early-2 was active in most or all pharyngeal cells and many non-pharyngeal cells in the head, suggesting that Early-2 could be a regulator of anterior or head-specific expression. The Late-2 element enhanced expression in the majority of pharyngeal cells, beginning at mid-embryogenesis, when the pharynx primordium initiates morphogenesis and differentiation (Figure 4M–4O; 1.5 fold stage; Sulston et al. 1983; Portereiko and Mango 2001). Late-2-dependent activity continued through embryogenesis but was relatively low or absent in larvae and adults. P-1 and P-2 functioned in pharyngeal cells after formation of the pharynx primordium, but both were relatively weak, variable enhancers compared to Early-1, Early-2, and Late-2 (Figure 4P–4U). Late-3 exhibited no enhancer activity. E1var, Early-3, and P-3 were not tested with this assay because of their similarity to other motifs (Early-1, CEH-22, and PHA-4, respectively). We conclude that five of the candidate elements can activate transcription broadly within the pharynx at distinct developmental stages.

**Figure 5:**
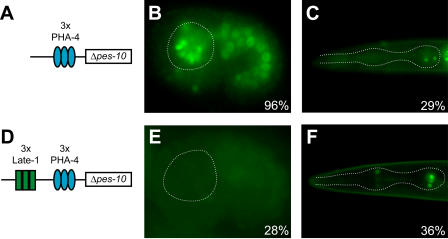
Late-1 Represses Early PHA-4-Dependent Expression Percent values indicate the percentage of transgenic animals exhibiting pharyngeal GFP expression. A reporter construct with three copies of a high-affinity PHA-4 site (TGTTTGC) upstream of the Δ*pes-10* promoter (A) expresses GFP in pharyngeal cells of most transgenic embryos (B) and roughly one-third of transgenic larvae (C). The addition of three copies of the Late-1 element from R07B1.9 (CCTTGGCGGCGC) to this transgene (D) drastically reduces expression in transgenic embryos (E) but has no observable effect on transgenic larvae (F). Dashed lines indicate the outline of the pharynx.

Tests of the Late-1 element revealed that this motif functions as a repressor rather than an enhancer (Figure 5). We demonstrated repression using a modified version of the Δ*pes-10* promoter in which three copies of the PHA-4 binding site were placed upstream of the Δ*pes-10* basal promoter to activate expression within the digestive tract (3×*pha-4*P::Δ*pes-10*::GFP). This construct expressed robustly in embryos and weakly in larvae and adults. To examine the Late-1 element for repressive activity, we inserted three copies of the Late-1 motif upstream of the PHA-4 sites. Remarkably, the presence of the Late-1 elements resulted in a marked decrease in embryonic GFP expression but had no effect on larval expression (Figure 5). Embryos that expressed GFP from the 3×Late-1::3×*pha-4*P::GFP reporter were late-stage embryos in which the pharynx was nearly or completely developed. Conversely, the Late-1 element was not active when tested for enhancer activity with the Δ*pes-10* promoter (data not shown). Thus, Late-1 functioned as a repressor of early gut expression. Late-1 may also function in cells outside of the digestive tract, but this aspect of regulation was not tested.

To characterize the temporal elements further, we examined their activity in endogenous pharyngeal promoters. We searched for examples of these motifs in predicted promoters that were conserved between C. elegans and C. briggsae to enrich for functionally relevant versions of motifs, rather than those that arose by chance alone. For Early-1 and Early-2, we tested the activity of these elements in the promoter of the early-onset pharyngeal gene K07C11.4. For Late-1, we tested the activity of this element in the promoter of the late-onset pharyngeal gene R07B1.9.

**Figure 6:**
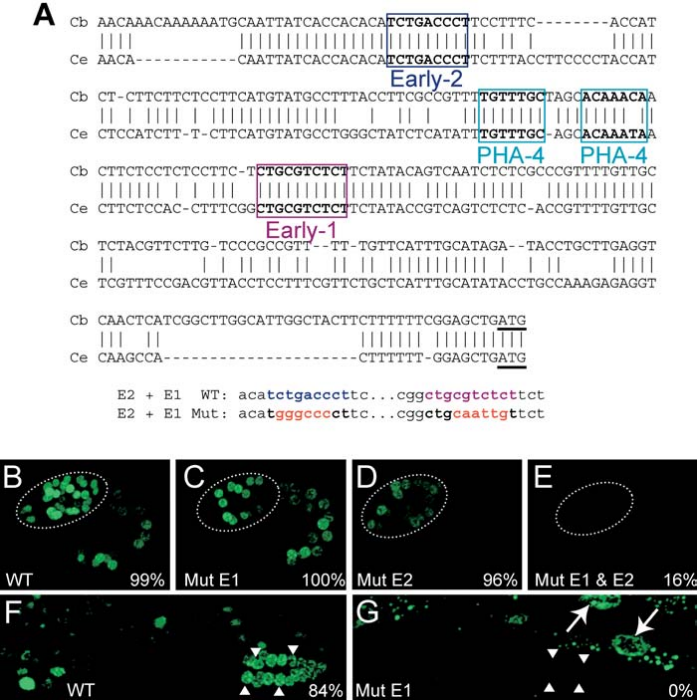
Early-1 and Early-2 Elements Are Required for K07C11.4 Expression (A) A portion of the promoter sequence of K07C11.4 from C. elegans (bottom) aligned with its ortholog from C. briggsae (top). Boxed regions show conserved predicted PHA-4 binding sites and Early-1 and Early-2 elements. Site-directed mutations that disrupt Early-1 and Early-2 (“E2 + E1 Mut”) are shown below their respective wild-type (“E2 + E1 WT”) sequence from K07C11.4. (B–E) Confocal images of mid-stage embryos expressing GFP under the control of the wild-type K07C11.4 promoter (B) or promoters with a mutation in Early-1 (C), Early-2 (D), or both Early-1 and Early-2 (E). Percentages are the fraction of transgenic embryos expressing GFP; the remainder of embryos do not express GFP. (F) Expression of the wild-type K07C11.4 reporter in a subset of somatic gonad cells in an L4 animal (arrowheads). (G) Mutation of the Early-1 element eliminates gonadal expression but does not strongly affect expression in other tissues, such as intestinal cells (arrows). Dashed lines indicate the outline of the developing pharynx.

We constructed a GFP reporter for K07C11.4 that faithfully recapitulated the expression observed by in situ hybridization experiments (Figure 6; Kohara 2001). K07C11.4::GFP was expressed from the stage of pharynx primordium formation to adulthood in the pharynx, midgut, and hindgut. K07C11.4::GFP was also active in the proximal somatic gonad of late larvae and adults. Alignment of K07C11.4 promoter sequences (500 bp upstream of the predicted ATG) between C. elegans and C. briggsae revealed stretches of conserved sequences. These regions contain two predicted PHA-4 binding sites (at −151 to −157 and −141 to −147, relative to the ATG), an Early-1 motif (−114 to −123), and an Early-2 motif (−217 to −225). The distal predicted PHA-4 site had a relatively high affinity for PHA-4 in vitro, consistent with this gene being expressed early in pharyngeal development (Gaudet and Mango 2002).

Early-1 and Early-2 elements were both positive regulatory elements for K07C11.4. Mutation of either Early-1 or Early-2 elements resulted in a decrease in the strength of reporter expression, but did not affect timing or cell-type specificity (Figure 6C, 6D, and 6G). Interestingly, the two elements made distinct contributions to the activity of the K07C11.4 promoter: mutation of the Early-2 element had a relatively moderate effect on all aspects of K07C11.4 promoter activity, while mutation of the Early-1 element had only a mild effect on expression in the digestive tract but completely abolished expression in the somatic gonad. Simultaneous mutation of both elements virtually extinguished expression in all tissues, suggesting that the two elements functioned additively for K07C11.4 expression (Figure 6E). We conclude that Early-1 and Early-2 elements are bona fide *cis*-regulatory elements for early gene expression in the pharynx and some non-pharyngeal tissues.

**Figure 7:**
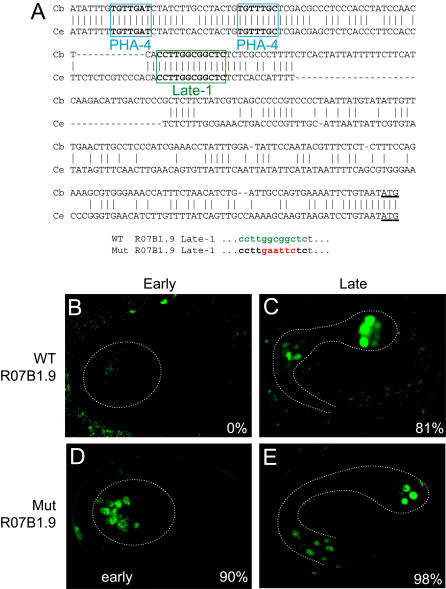
The Late-1 Element Negatively Regulates R07B1.9 (A) A portion of the promoter sequence of R07B1.9 from C. elegans (bottom) aligned with its ortholog from C. briggsae (top). Boxed regions show conserved predicted PHA-4 binding sites and a Late-1 element. The site-directed mutation that disrupts Late-1 (“Mut”) is shown below the respective wild-type sequence from R07B1.9. (B and C) Confocal images of representative early and late embryos expressing GFP under the control of the wild-type R07B1.9 promoter. (D and E) Confocal images of representative early and late embryos expressing GFP under the control of the R07B1.9 promoter with a mutation in the Late-1 element. Note the early activation of R07B1.9 when Late-1 is inactivated. Dashed lines indicate the outline of the developing pharynx.

To assess Late-1 activity, we constructed a reporter for R07B1.9 that reproduced the endogenous pattern of expression (from in situ hybridizations; Kohara 2001; Figure 7). R07B1.9 was activated during the terminal stages of pharynx development, (the 3-fold stage; Sulston et al. 1983), and its expression was maintained throughout the life of the animal (Figure 7B and 7C). The promoter of R07B1.9 contained two predicted PHA-4 binding sites (−243 to −249 and −221 to −227) and one Late-1 element (−170 to −180), all of which were conserved in C. briggsae (Figure 7A).

The Late-1 element functioned as a repressor for R07B1.9, as predicted. Elimination of the Late-1 element led to precocious expression 2–3 h earlier than the wild-type (Figure 7D and 7E). GFP was first visible when the pharynx primordium was formed and continued throughout the life of the animal. The mutant reporter was expressed in the same cells and at the same approximate strength as the wild-type, suggesting that disruption of the Late-1 element specifically affected the onset of gene expression. We conclude that six of the motifs (Early-1, Early-2, Late-1, Late-2, P-1, and P-2) display activity in vivo and that at least three of these contribute to temporal regulation of endogenous pharyngeal genes.

### Temporal Elements and PHA-4 Sites Determine Onset of Expression

Our analyses suggested that the Early and Late elements function in combination with PHA-4 sites to modulate the onset of pharyngeal gene expression. We performed three tests to address the generality of this model. First, we surveyed the Ph-E and Ph-L gene clusters to determine the configurations of the five regulatory elements (Early-1, Early-2, Late-1, Late-2, and PHA-4 sites) within endogenous promoters. Second, we constructed synthetic promoters to examine the interplay between the temporal elements and PHA-4 binding sites. Third, we combined our observations from the synthetic and endogenous promoters to search the genome for genes containing similar configurations of the five *cis*-regulatory motifs.

#### Temporal regulatory elements in Ph-E and Ph-L genes

We examined Ph-E and Ph-L genes to see if their promoters contained distinct combinations of Early, Late, and PHA-4 sites (Table 4; Table S4). For PHA-4 sites, we also examined the predicted affinity of these sites because the affinity of PHA-4 for its binding sites influences the onset of expression (Gaudet and Mango 2002). For Ph-E and Ph-L, 22/33 and 19/33 genes, respectively, had temporal elements and predicted PHA-4 sites that were conserved in their C. briggsae orthologs. Interestingly, particular combinations of elements appear to be associated with Ph-E or Ph-L genes (Table 4). For example, combinations of Early elements with PHA-4 sites of predicted high or medium affinity are far more frequent in Ph-E genes than in Ph-L genes (11/22 [50%] versus 2/19 [11%], respectively), suggesting that this configuration of elements promotes early pharyngeal expression. In contrast, combinations of Late elements with PHA-4 sites of varying affinity are frequent in Ph-L genes (9/19 [47%]) but do not occur at all in Ph-E genes. In addition, promoters with only low-affinity PHA-4 sites together with any temporal elements are more common in Ph-L than in Ph-E genes (4/19 [21%] versus 2/22 [9%], respectively). These trends suggest that late pharyngeal gene expression is promoted by the combination of either Late elements with any PHA-4 site, or low-affinity PHA-4 sites with any temporal elements. We conclude that expression of at least half of Ph-E and Ph-L genes can be accounted for by a combination of PHA-4 sites and temporal elements.

**Table 4:**
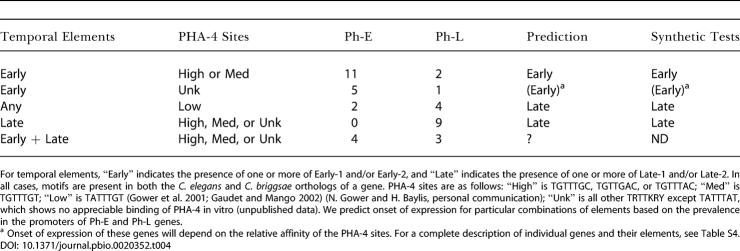
Combinations of Temporal Elements and PHA-4 Sites Predict Onset of Pharyngeal Gene Expression

For temporal elements, “Early” indicates the presence of one or more of Early-1 and/or Early-2, and “Late” indicates the presence of one or more of Late-1 and/or Late-2. In all cases, motifs are present in both the C. elegans and C. briggsae orthologs of a gene. PHA-4 sites are as follows: “High” is TGTTTGC, TGTTGAC, or TGTTTAC; “Med” is TGTTTGT; “Low” is TATTTGT (Gower et al. 2001; Gaudet and Mango 2002) (N. Gower and H. Baylis, personal communication); “Unk” is all other TRTTKRY except TATTTAT, which shows no appreciable binding of PHA-4 in vitro (unpublished data). We predict onset of expression for particular combinations of elements based on the prevalence in the promoters of Ph-E and Ph-L genes

^a^ Onset of expression of these genes will depend on the relative affinity of the PHA-4 sites. For a complete description of individual genes and their elements, see Table S4

#### Synthetic promoters recapitulate temporal expression

The combination of elements observed in natural promoters suggests that a gene with a high-affinity PHA-4 site and an Early element will be expressed early in development, while a gene with a high-affinity PHA-4 site and a Late element will be expressed later in development. From the existing Ph-E and Ph-L promoters, however, the output of certain promoter configurations cannot be reliably predicted. For example, when a low-affinity PHA-4 site is paired with an Early element, is the gene activated early or late in development? A low-affinity PHA-4 site in the presence of an Early element might be expected to activate expression relatively late, as determined by the binding of PHA-4 to the low-affinity site, or early, if the Early element potentiates earlier expression. To test these ideas, we constructed artificial promoters within the context of the Δ*pes-10* promoter and examined their expression patterns.

**Figure 8:**
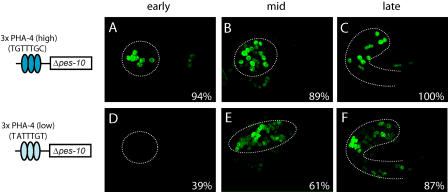
High-Affinity PHA-4 Sites Activate Pharyngeal Expression Earlier Than Low-Affinity Sites Percent values indicate the percentage of transgenics exhibiting pharyngeal GFP expression. Dashed lines indicate the outline of the developing pharynx. (A–C) A reporter construct with three copies of a high-affinity PHA-4 site (TGTTTGC) upstream of the Δ*pes-10* promoter reproducibly activates pharyngeal expression from the time of pharynx primordium formation (“early”) through embryogenesis. (D–F) A reporter construct with three copies of a low-affinity PHA-4 site (TATTTGT) upstream of the Δ*pes-10* promoter activates pharyngeal expression from the time of attachment of the pharynx to the mouth (“mid”) through embryogenesis.

We first investigated the behavior of the high- and low-affinity PHA-4 binding sites in synthetic promoters, because the configuration of Ph-E and Ph-L promoters together with previous work (Gaudet and Mango 2002) suggested that onset of expression was influenced by the affinity of PHA-4 sites. To test this idea, we compared the activity of three copies of either a high- or low-affinity PHA-4 site placed in front of the Δ*pes-10* promoter (Figure 8). As observed previously (Okkema and Fire 1994), three copies of a high-affinity PHA-4 site were sufficient to activate pharyngeal expression beginning early in development, prior to formation of the pharynx primordium (*n =* 34/36). In contrast, three copies of a low-affinity PHA-4 site were sufficient to activate pharyngeal expression later in development. Early expression was observed significantly less frequently with the low-affinity constructs than with the high-affinity constructs (*n* = 28/71). Notably, the strength of expression of both constructs was comparable in late embryos. We conclude that promoter activation depends on the affinity of PHA-4 for its binding sites and cannot be compensated for by multimers. This finding agrees with data that show FoxA factors bind DNA as monomers, not cooperatively (Clark et al. 1993).

We next tested synthetic promoters containing a single PHA-4 site (either high or low affinity) with a single temporal element (either Early-1 or Late-2) to determine how these elements behave in combination. The synthetic promoter constructs differed from our initial Δ*pes-10*::GFP reporter survey in that they resembled the configuration of endogenous promoters. While temporal elements and PHA-4 sites do not appear to exhibit conserved spacing or order within pharyngeal promoters, each element typically occurs in one or two copies per promoter. We therefore constructed reporters in which one copy of either an Early-1, Early-2, or Late-2 element was paired with one copy of either a high- or low-affinity PHA-4 site, separated by approximately 100 bp.

**Figure 9:**
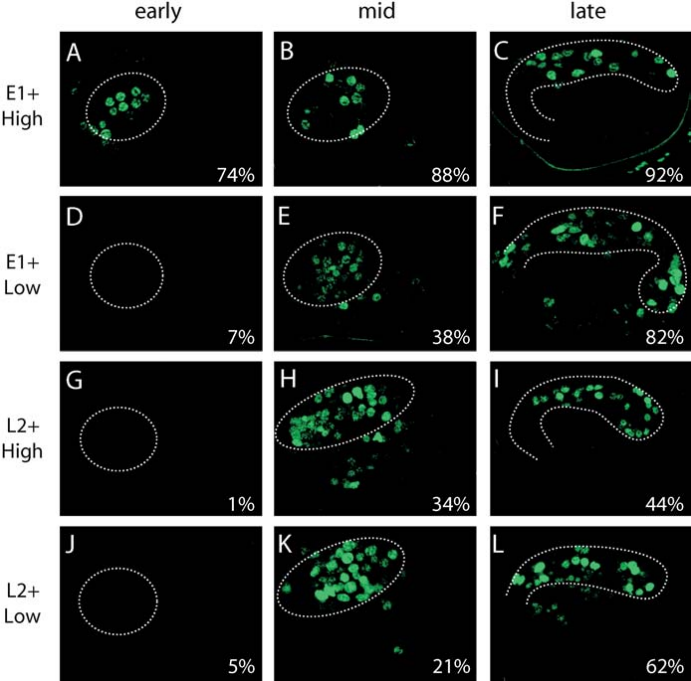
Temporal Elements Combined with PHA-4 Sites Regulate the Onset of Pharyngeal Expression “Early,” “mid,” and “late” are as defined in Figure 3. E1, Early-1; L2, Late-2; High, high-affinity PHA-4 site (TGTTTGC); Low, low-affinity PHA-4 site (TATTTGT). Percent values indicate the percentage of transgenics exhibiting pharyngeal GFP expression; the remainder of embryos do not express GFP. A reporter construct with one copy of the Early-1 element and one copy of a high-affinity PHA-4 site is expressed in “early” to “late” embryos (A–C). In contrast, a reporter with one copy of the Early-1 element and one copy of a low-affinity PHA-4 site is not consistently expressed until the “mid” to “late” stages (D–F). Reporters with one copy of Late-2 and one copy of either a high-affinity (G–I) or low-affinity (J–L) PHA-4 site are expressed in “mid” and “late” stage embryos. Dashed lines indicate the outline of the developing pharynx.

The onset of expression for artificial promoters containing an Early-1 site depended on the relative affinity of the accompanying PHA-4 site (Figure 9), as predicted. The promoter with a high-affinity PHA-4 site was reliably expressed at all embryonic stages examined, including early pharyngeal development (*n* = estimated number of transgenic embryos scored = 17/23; see Materials and Methods for an explanation of the estimate). In contrast, the promoter with the low-affinity PHA-4 site was consistently expressed in late embryos, but only infrequently in earlier stages (*n* = 4/58). Because both artificial promoter constructs activated expression comparably in late embryos, we conclude that the differences in early expression reflect a genuine difference in the onset of expression between constructs rather than a difference in the strength or penetrance of expression. These data support a model in which the Early-1 element is able to mediate early pharyngeal gene expression but where the onset of gene expression is ultimately limited by the relative affinity of the PHA-4 binding site. We similarly tested Early-2 together with a high-affinity PHA-4 site, but did not observe any expression above background, suggesting that this combination or configuration of sites was not sufficient to activate pharyngeal expression.

Artificial promoters containing the Late-2 element were not expressed until mid-embryonic to late embryonic development, regardless of the relative affinity of the PHA-4 site (Figure 9; *n =* 1/73 early embryos for constructs with a high-affinity PHA-4 site, *n =* 2/37 early embryos for constructs with a low-affinity site). Because the Late-2 element behaved as an enhancer, we hypothesize that the factor or factors that act through Late-2 are not available until late in development and that their absence in early development results in delayed expression of Late-2 dependent genes.

#### Genome searches identify additional pharyngeal genes

The temporal motifs offered an opportunity to discover new pharyngeal genes based solely on predicted *cis*-regulatory sites. We searched the C. elegans and C. briggsae genomes for conserved occurrences of the temporal motifs, together with PHA-4 sites, to determine whether combinations of these sites were predictive for pharyngeal genes. We required the presence of three elements within a 500-bp stretch because Ph-E and Ph-L averaged a total of 3.7 elements in their promoters and because artificial promoters with only two elements generated weaker expression than endogenous promoters (Figure 9). We chose a stringent threshold score for each element that maximized the ability to distinguish elements in control runs of early versus late pharyngeal genes (see Materials and Methods). Although these thresholds reduced the number of elements identified in the positive control set (e.g., the Early-1 element in Ph-E promoters) they also dramatically lowered the false positive rate (e.g., the Early-1 element in Ph-L promoters).

The combination of Early-1, Early-2, and PHA-4 sites provided a powerful approach to predict early pharyngeal genes. Of 40 genes with conserved copies of all three elements, 20 had available expression data, and 70% of these were expressed in the pharynx (14 genes; Table S5). In contrast, of 194 randomly selected genes, 57 had expression data and only 12% (7/57) of these were expressed in the pharynx (data not shown), indicating that the results of the genomic searches are significantly enriched for pharyngeal genes (*p* = 6 × 10^−7^). Furthermore, of the 14 pharyngeal genes identified by the genomic search, 86% (12/14) were expressed early in pharyngeal development, as predicted. The 14 pharyngeal genes included ten genes not identified in our microarray experiments, reflecting the power of bioinformatics in predicting gene expression.

Given the possibility that E1var is a functional temporal element, we performed genomic searches for genes that contain conserved E1var plus Early-2 plus PHA-4 elements together within 500 bp of their predicted start codon. This search identified 120 genes with some overlap with the Early-1 search output. Of these 120 genes, 53 had expression data (Table S5), and 47% of these (25/53) were expressed in the pharynx, a significant enrichment compared to the random set (*p* = 6 × 10^−5^), suggesting that, like Early-1, E1var in combination with other sites is predictive for pharyngeal expression. Of these pharyngeal genes, 70% (16/23; onset of expression not determined for two genes) were expressed early, as predicted. Combining the results of the Early-1 and E1var searches, we identified a total of 35 pharyngeal genes, 73% of which (24/33) were expressed early.

For late expression, 61 genes had conserved Late-1, Late-2, and PHA-4 sites, and these were also enriched for pharyngeal genes. Thirty of these genes had expression data, of which 33% (ten genes) were pharyngeally expressed (Table S5), showing significant enrichment compared to the random gene set (*p* = 0.02). Strikingly, the Late elements accurately predicted the timing of expression in the pharynx, as all ten pharyngeal genes were expressed late in pharyngeal development. Considering the Early and Late genomic searches together, we identified 45 pharyngeal genes, 36 of which were not present in our microarray positives. Furthermore, the onset of expression of these 45 pharyngeal genes was predicted with 88% overall accuracy. There are three different reasons for our microarray experiments not identifying the 36 new pharyngeal genes: (1) some genes are expressed in both pharyngeal and non-pharyngeal cells and would not be significantly enriched in the *par-1* versus *skn-1* samples, (2) some genes are expressed post-embryonically and would not have been present in the embryonic samples used for the microarray experiments, and (3) some genes scored just below the 2-fold enrichment threshold for inclusion in our positive set.

## Discussion

The advent of microarray technology and bioinformatics has provided a powerful tool to dissect the transcriptional regulatory circuits that guide complex developmental processes. We have analyzed pharynx organogenesis and defined novel regulatory elements that contribute to temporal gene expression. Our screens differ from previous examples of bioinformatic analyses in that the regulatory motifs were identified in unbiased searches of promoter sequences and subsequently tested for biological activity. We used three assays to demonstrate a function in vivo. First, we tested whether the regulatory elements were necessary for expression of native pharyngeal genes. Second, we determined whether they were sufficient for pharyngeal expression from synthetic promoters. And third, we did genome-wide searches based on these elements, a process that identified 19 new pharyngeal genes, of which 88% displayed the expected onset of expression. The *cis*-regulatory motifs discovered here, combined with the PHA-4 binding site, establish a regulatory network that can account for the timing of activation of at least half of C. elegans pharyngeal genes.

### A Model for Temporal Control of Pharyngeal Gene Expression

We previously proposed a model in which the relative affinity of PHA-4 for its binding sites controls the onset of pharyngeal gene expression. Because PHA-4 protein levels increase during development, we proposed that initially PHA-4 levels are low and only high-affinity sites are sufficiently occupied by PHA-4 to result in gene activation. As PHA-4 levels increase over time, lower-affinity sites also become occupied, leading to the expression of those genes. In addition, binding site affinity likely affects PHA-4 occupancy even at stable PHA-4 concentrations. The degree of PHA-4 occupancy would likely alter the probability of productive transcription of a target gene. However, the affinity model could not explain all temporal expression because some late-expressed pharyngeal genes had high-affinity PHA-4 binding sites (e.g., *myo-2* and R07B1.9).

In this study, we have identified additional regulatory elements that establish the onset of pharyngeal gene expression in combination with PHA-4. We suggest that the affinity of PHA-4 for its binding site determines the earliest possible time of pharyngeal gene expression but that other factors must be present for a promoter to be active. The Early elements likely represent binding sites for transcription factors that are available throughout embryonic pharyngeal development. In contrast, the Late-2 element is probably recognized by a transcription factor that is not available until midway through pharynx development, thereby delaying expression of these pharyngeal genes regardless of the quality of the PHA-4 binding sites. We note that our searches were restricted to genes with similar onset of expression, in order to identify temporal regulatory elements, and there may be yet more undiscovered elements that control temporal expression. For example, an element that confers cell-type-specific expression could also control onset of expression according to the availability of the relevant binding factor. Consistent with the idea of additional motifs, the synthetic promoters with two enhancer elements were functional but weak activators of expression compared to native promoters, which likely contain more regulatory motifs (e.g., *myo-2*).

In addition to enhancers of pharyngeal expression, we identified one negative regulatory element, Late-1, that is dominant to PHA-4 in early pharynx development. The proximal PHA-4 consensus site in the R07B1.9 promoter is predicted to be a high-affinity binding site, suggesting that R07B1.9 should be expressed early in pharynx development, not late as is observed. Mutation of the Late-1 element enabled the R07B1.9 promoter to fire at earlier developmental stages. We hypothesize that some factor binds to Late-1 and represses expression in early pharynx development. Subsequently, the Late-1 factor is presumably inactivated or downregulated to permit gene expression.

The elements characterized here can account for the temporal expression patterns of many but not all known pharyngeal genes. One possible reason for this is that we do not yet have sufficient information to identify all functionally relevant occurrences of these elements. For example, some occurrences of the temporal elements may not be biologically meaningful, thereby generating false positives, while others may be hard to identify because of sequence heterogeneity or placement within *cis*-regulatory sequences. Our analyses initially focused on regions within 500 bp of the predicted start codon of genes, but we further searched −501 to −1,000 upstream of the ATG for Ph-E and Ph-L genes whose expression could not be accounted for by elements in the region of −1 to −500. However, these extended searches did not find any conserved elements that could further account for onset of expression (data not shown). Another possibility is that regulatory elements may be found within introns or the 3′ UTR (Shibata et al. 2000; Marshall and McGhee 2001; Gaudet and Mango 2002; Kirouac and Sternberg 2003). Consistent with this observation, we find that 40% of the Ph-E and Ph-L genes show significant conservation of intron sequence between C. elegans and C. briggsae (data not shown). Thus, some of the genes whose onset of expression cannot be accounted for in our analyses likely have larger and more complex regulatory regions than the 500-bp window we used. Nonetheless, this limited sequence window allowed us to account for the onset of expression of roughly half of the Ph-E and Ph-L genes.

An interesting feature of the Early-1 element is that it is necessary for the expression of K07C11.4 in the proximal somatic gonad, as well as in the pharynx. Some, though not all, genes containing conserved Early-1 elements are expressed in the proximal somatic gonad (e.g., F30H5.3, F47D12.7, and *ncr-1*; Kohara 2001), consistent with Early-1 being necessary but not sufficient for gonadal expression. PHA-4 is also expressed in the somatic gonad, including the proximal region (Azzaria et al. 1996; Kalb et al. 1998). The finding of multiple genes expressed in the two tissues under control of Early-1 and possibly PHA-4 suggests the presence of shared regulatory mechanisms in the two organs. Both the pharynx and proximal somatic gonad are epithelial tubes that connect to the external environment and as such may have conserved features that are regulated by some of the same factors. The role of PHA-4 in the gonad has not been carefully analyzed, but PHA-4 is critical for proper gonad development post-embryonically (Ao et al. 2004).

### Candidate *trans-*Acting Factors

The identification of new pharyngeal regulatory elements provides an entry point for identifying the relevant transcription factors that bind these sequences. We searched the TRANSFAC database and available literature for possible matches to the temporal elements identified here (Knuppel et al. 1994; Matys et al. 2003). While we did not discover obvious candidate factors for the relevant transcription factors, we did find intriguing similarities between our elements and known transcription factor binding sites. The Early-1 element resembles the recognition sequence for *Drosophila* ZESTE and GAGA (YGAGYG and GAGAG, respectively; Benson and Pirrotta 1987; Omichinski et al. 1997). However, no clear ortholog of either gene exists in C. elegans. Early-2 resembles a hemi-site for nuclear hormone receptors, but the identification of specific candidate factors is complicated by the existence of 284 predicted nuclear-hormone-receptor-encoding genes in C. elegans (Maglich et al. 2001). The Late-1 element is a GC-rich sequence that resembles a Sp-factor binding site and behaves as a negative regulator of early expression. C. elegans has four Sp-like homologs (Zhao et al. 2002), one of which is a predicted pharyngeal gene (F45H11.1). However, F45H11.1(RNAi) did not affect expression of the Late-1-regulated gene R07B1.9 (data not shown). The Late-2 element (TTTTTCC) most closely resembles a Dorsal/Rel-homology domain binding site (KGGWWWWCCC; Matys et al. 2003), but there are no C. elegans Rel-homology domain proteins. Given the lack of other obvious candidate factors for the temporal elements, molecular or genetic screens will be required to identify the relevant transcription factors.

### Elements and Regulatory Modules

Recent studies of transcription factor binding sites have revealed cases of multimers of a single site being important for expression (Berman et al. 2002; Markstein et al. 2002; Yoo et al. 2004) and cases of entire modules of elements being conserved features of some promoters (Senger et al. 2004). By contrast, we see little evidence of conserved spacing, order, or organization of our elements within pharyngeal promoters. Genomic searches for genes with multiple copies of a single element yielded few pharyngeal genes, suggesting that the temporal elements and PHA-4 sites typically act in single copy (data not shown).

The analysis of PHA-4 targets suggests two strategies of transcriptional control. We propose that a minority of target genes respond consistently to the presence of PHA-4. For example, K07C11.4, T05E11.3, and M05B5.2 are active broadly throughout the pharynx and in other cells that express PHA-4, such as the gonad or rectum (Gaudet and Mango 2002). These promoters contain four or more predicted PHA-4 binding sites, including at least one high-affinity site. The density of high-quality PHA-4 binding sites may promote activation of these genes whenever PHA-4 is present. This strategy may have been adopted by other transcription factors. For example, likely target genes of the Notch effector CSL (also known as CBF1/RBP-Jκ_e_, Su(H), and LAG-1) have been discovered in C. elegans and *Drosophila* (Christensen et al. 1996; Rebeiz et al. 2002; Yoo et al. 2004). Many of these genes encode components of the Notch signaling pathway that function upstream of CSL and would therefore be expected to respond broadly to CSL as part of a regulatory feedback loop. Intriguingly, these genes contain multiple copies (e.g., 15–25) of the CSL binding site consensus, which may facilitate CSL-mediated activation (Christensen et al. 1996).

On the other hand, the majority of pharyngeal genes respond to PHA-4 in some cellular environments but not others (Okkema and Fire 1994; Gaudet and Mango 2002). These genes appear to depend on a second regulatory strategy, in which combinations of elements synergize, with no individual element sufficient for transcription. These promoters typically carry one or two copies of any given element, as observed for the *myo-2* promoter (Okkema and Fire 1994; Thatcher et al. 2001; Gaudet and Mango 2002; Ao et al. 2004). Similarly, CSL factors have target genes that are activated in only a subset of tissues, and these contain only one or a few high-quality binding sites (for example, *vestigial* or *D-pax2;* Kim et al. 1996; Flores et al. 2000).

The combinatorial mode of regulation relies on the relatively poor transcriptional activity of the individual factors. For example, a single Early-1 or PHA-4 binding site cannot activate expression of Δ*pes-10*::GFP. Moreover, PHA-4 can transform some cells towards a pharynx fate, but is not as potent as other developmental regulators such as *end-1* (Horner et al. 1998; Kalb et al. 1998; Zhu et al. 1998). Ectopically-expressed END-1 can transform the entire embryo into midgut (Zhu et al. 1998). Accordingly, END-1 is expressed briefly within midgut precursors, where it likely activates a uniform panel of downstream targets (Zhu et al. 1997). We suggest that the inherent activity of a transcriptional regulator coupled with promoter architecture defines the range of target genes available to a developmental transcription factor.

The combinatorial mode of regulation exhibited by PHA-4 provides the organism with transcriptional flexibility in two ways. First, it provides a mechanism for selectivity for Fox transcription factors. The worm genome encodes fifteen Fox transcription factors, and these proteins regulate diverse biological activities such as cell fate specification, longevity, and cell migration (Miller et al. 1993; Ogg et al. 1997; Horner et al. 1998; Kalb et al. 1998; Nash et al. 2000; Sarafi-Reinach and Sengupta 2000; Hope et al. 2003). Combinatorial regulation affords the animal with a means to distinguish different target genes for different Fox proteins, all of which share a similar DNA binding domain. Second, the combinatorial strategy enables PHA-4 to play a broad role in the pharynx. PHA-4 is activated at the earliest stages of organogenesis in all pharyngeal cells, where it is required to specify different pharyngeal cell types. PHA-4 continues to be expressed throughout the life of the animal, where it likely controls pharyngeal function and growth. These different functions presumably reflect different target genes activated in different cell types or during different developmental stages. FoxA factors in other animals exhibit long-term expression and, like PHA-4, are poor inducers of cell fate when expressed ectopically (Sasaki and Hogan 1994). FoxA target genes such as albumin require additional factors for activation, and FoxA promoter association is not sufficient for transcription (Zaret 1999). These data indicate that mammalian FoxA proteins likely rely on a transcriptional strategy similar to that of worms.

## Materials and Methods

### 

#### Identification of genes selectively expressed in the pharynx

C. elegans strains KK822 (*par-1(zu310) IV;* Kemphues et al. 1988) and EU1 (*skn-1(zu67)/DnT1 IV;V;* Bowerman et al. 1992) were grown in liquid culture with OP50 as a food source, synchronized, and harvested. For KK822, we shifted synchronized homozygotes to the restrictive temperature (25 °C) and isolated embryos by hypochlorite treatment (Sulston and Hodgkin 1988). For EU1, we grew synchronized animals and plated young adults. *skn-1/DnT1* worms are uncoordinated, while *skn-1* homozygotes are non-uncoordinated, allowing us to enrich for *skn-1* homozygotes using a plate crawling assay. To synchronize, we performed a first round of hypochlorite embryo isolation and then allowed embryos to hatch overnight in liquid culture lacking food to obtain L1 larvae. We then added food to the cultures and grew the animals for 2–3 d to age them. For *par-1,* animals were aged to young adults, and embryos were collected from these by hypochlorite treatment. For the *skn-1/DnT1* strain, animals were aged to L4 and then transferred to large plates for the crawling assay and later collected as young adults for embryo isolation. Collected embryos from either strain were aged another 3–6 h in small liquid cultures to ensure that *par-1* and *skn-1* embryos were at approximately the same stage of development, as determined by examining a sample of embryos under the light microscope. To extract total RNA from isolated C. elegans embryos, embryos pellets were frozen in microfuge tubes, crushed with a plastic pestle, and resuspended in RNA extraction buffer (1% lauroyl sarcosine, 0.1 M Tris base, 0.1 M NaCl, and 20 mM EDTA), followed by several rounds of phenol:chloroform extraction and ethanol precipitation (Horner et al. 1998; Gaudet and Mango 2002). Once-selected poly-A+ RNA was purified using the PolyTract Isolation Kit (Promega, Madison, Wisconsin, United States). *par-1* cDNAs were labeled with Cy3, and *skn-1* cDNAs were labeled with Cy5. Labeled cDNA was prepared from 5 μg of poly-A+ RNA by the Huntsman Cancer Institute Microarray Core Facility. Construction and probing of the microarrays was as described by Reinke et al. under the auspices of the Kim lab (Reinke et al. 2000).

#### Analysis of microarray data

We performed two microarray experiments using microarrays containing 62% of the C. elegans genome (“partial arrays,” experiments PS1 and PS2) and three experiments using microarrays containing 94% of the genome (“full arrays,” experiments PS3, PS4, and PS5). Our previous microarray experiments (PS1–PS3) detected 242 positives, corresponding to 227 genes (Gaudet and Mango 2002).

To extend the identification of candidate pharyngeal genes, we included data from PS4 and PS5. In this case, we selected genes that had an average log_2_(*par-1/skn-1*) ≥ 1.00 in PS1–PS5 and were expressed above background in three of five experiments. In this selection, we observed considerable background of non-pharyngeal genes, primarily genes with maternally contributed transcripts or expression in pre-gastrulation embryos. We hypothesize that this background is the result of our *par-1* embryos being harvested at an earlier stage than our *skn-1* embryos in experiments PS4 and PS5. Because PS1–PS3 do not appear to exhibit this difference in staging, we applied an additional selection criterion to our data, requiring that all positives have a log_2_(*par-1/skn-1*) ≥ 0.58 on PS1, PS2, or PS3. This threshold reduces the inclusion of non-pharyngeal genes identified by PS4 and PS5 and identifies a total of 241 microarray positives. We further subtracted probable maternal genes from this set of positives using the C. elegans expression map of Kim et al. (2001), leaving us with 118 microarray positives corresponding to 112 new candidate pharyngeal genes. As a validation of these experiments, we note that among these 112 new genes are 44 genes with known expression patterns, and 33/44 (75%) of these are pharyngeally expressed. This fraction of pharyngeal genes is comparable to the fraction of pharyngeal genes present in our set of positives from PS1–PS3 (81/97 [84%]).

#### Examining sequence conservation

C. elegans and C. briggsae upstream and downstream sequences were extracted from the genome using the Ensembl EnsMart tool (Kasprzyk et al. 2004). For four genes, ENSEMBL did not provide orthologous C. briggsae sequences. In these cases we used the Intronerator Tracks Display (Kent and Zahler 2000) to obtain the C. briggsae sequence. Sequences were then aligned and visualized by VISTA (Mayor et al. 2000). We scored as conserved those regions of DNA that had 75% or greater identity over 50 or more base pairs. Alignments in Figures 6 and 7 were obtained from the Tracks Display feature of Intronerator (Kent and Zahler 2000).

#### Motif searches using Improbizer

We used the Improbizer program (Ao et al. 2004; available at http://www.soe.ucsc.edu/approximately/kent/improbizer/), which employs a variation of the expectation maximization algorithm (Bailey and Elkan 1994), to search for motifs. Improbizer was able to find expected regulatory elements (i.e., motifs that resemble PHA-4 and CEH-22 binding sites) in our promoter sequences, while other algorithms did not. Improbizer can be configured to simultaneously search for motifs on both strands of DNA, search for more than a single occurrence of a motif on each DNA sequence, and use a separate set of sequences for a background model. The source code for the Improbizer is freely available, and is the definitive reference for the details of the algorithm.

In using Improbizer to search the Ph-E and Ph-L gene sets for possible regulatory motifs, we initially searched for motifs occurring once per sequence, with an initial scan through the first five sequences entered. For each gene set we ran searches three or more times, varying the order of input for genes in each run. Motifs presented here were obtained with searches for a motif size of six. Searches for motifs of larger sizes (8–20 bases) recurrently found variations of the motifs presented here. Other parameters of Improbizer were used at their default settings. For background sequences, we used three different sets of sequences (foreground sequence, and upstream sequences from each of two gene groups defined by Kim et al. [2001]: neuronal genes and carbohydrate metabolism genes) and obtained similar output matrices in all cases. All matrices presented here were obtained using the input sequence (foreground) as the background. This mixing was performed to prevent output being biased towards sequence motifs found in only the first few input genes. The motifs presented here were reproducibly obtained independent of the order of input sequences.

As an initial screen for motifs that were over-represented in our test sets, we performed control runs in which the input gene sequence was randomized and searched. All motifs presented here obtained Improbizer scores greater than the scores of ten or more control runs.

#### Identification of motifs using Cluster-Buster

Cluster-Buster finds the best possible occurrence of a motif (or motifs) in a given sequence and therefore requires the establishment of a threshold score to determine which occurrences are likely to be meaningful (Frith et al. 2003). We established threshold scores for our motifs that maximized the ratio of “hits” in a positive versus a negative group, with a “hit” defined as a gene that contained an occurrence of a motif above the threshold. For example, we chose a threshold score for Early-1 that gave the greatest ratio of Ph-E hits to Ph-L hits. These thresholds were then applied to searches of C. elegans Ph-E and Ph-L genes and their C. briggsae orthologs to determine which genes contained Early and Late elements in both C. elegans and C. briggsae. Position within the promoter was not required for a motif to be considered “conserved.” For Tables 3 and S4, the following parameters were used: the cluster score threshold *(C)* and gap parameter *(g)* for all motifs were zero and 35, respectively. The motif threshold *(m)* used for Early-1, Early-2, E1var, and Late-1 was six, for Late-2 was seven, and for PHA-4 was five. The random gene set referred to in Table 3 was generated using a random number generator to select 200 genes from a complete list of all predicted C. elegans genes. Duplicates or splice isoforms of a gene were collapsed to a single selection, resulting in a total of 194 genes.

For the genome searches, we searched for genes that contained a set of elements (e.g., Early-1 plus Early-2 plus PHA-4) within the first 500 bp of upstream sequence in both C. elegans and C. briggsae. We applied more stringent thresholds to the elements for these searches, to minimize the identification of non-pharyngeal genes. We optimized the Early element thresholds by searching the genome to identify known Ph-E and Ph-L genes and selecting the search parameters that maximized the ratio of Ph-E/Ph-L genes identified. The Cluster-Buster (Frith et al. 2003) parameters for Early-1 plus Early-2 combined were *C* = 3.5, *m* = 6, and *g* = 35. The PHA-4 parameters were *C* = 1.9, *m* = 6, and *g* = 35. For E1var plus Early-2 combined the parameters were *C* = 1, *m* = 5.5, and *g* = 35. The PHA-4 parameters were *C* = 2, *m* = 6, and *g* = 35. Using these thresholds, our genomic searches identified four known Ph-E genes but no Ph-L genes. The same approach was used to optimize parameters for genome searches with the Late elements, maximizing the ratio of Ph-L/Ph-E genes identified. The parameters for Late-1 plus Late-2 combined were *C* = 2, *m* = 6, and *g* = 35. The PHA-4 parameters were *C* = 2.5, *m* = 6, and *g* = 35.

#### Construction of plasmids

To construct transcriptional reporters, we amplified promoter sequences from genomic N2 DNA using gene-specific primers that contained restriction endonuclease sites to facilitate cloning. PCR products were cloned into the vector pAP.10, which carries a GFP::HIS2B translational fusion, resulting in a nuclear-localized GFP (Gaudet and Mango 2002). This cloning strategy removed the *pes-10* promoter sequence present in pAP.10.

Enhancer constructs and synthetic promoters were built using synthetic oligonucleotides that were cloned into pAP.10, upstream of the Δ*pes-10* promoter fragment. Clones were verified by restriction digests and sequencing. For the triplicate enhancer sequences, we used the following insert sequences (sequences of the individual motifs are underlined; periods show spacing of elements in Late-1): Early-1, AGAGACGCAGATTAGAGACGCAGATTAGAGACGCAGATT; Early-2, T AACTGACCGTCTTAACTGACCGTCTTAACTGACCGTCT; Late-1, CTTGGCGGCGCC.CTTGGCGGCGCC.CTTGGCGGCGCC; Late-2, CTCTTTTTCCCACTCTTTTTCCCACTCTTTTTCCCA; Late-3, ACTCTCGGAATCACTCTCGGAATCACTCTCGGAATC; P-1, TTGCTCACCTAATTGCTCACCTAATTGCTCACCTAA; P-2, TTTCTTCCAAATTTTCTTCCAAATTTTCTTCCAAAT; PHA-4 (high), CTACTGTTTGCCCCTACTGTTTGCCCCTACTGTTTGCCC; and PHA-4 (low), CTACTATTTGTCCCTACTATTTGTCCCTACTATTTGTCC.

For the synthetic promoters, individual Early-1 or Late-2 sites were cloned in to the SphI and SalI sites of pAP.10, and individual PHA-4 sites were cloned in to the NheI and NsiI sites of pAP.10. Fragments from these single-site constructs were ligated to generate the constructs containing one temporal site and one PHA-4 site, with the sites separated by 95 bp of pAP.10 sequence. The sequences of the synthetic regions of these constructs were as follows (individual motifs are underlined): Early-1 plus PHA-4 (high), GCATGCTCGAGAGACGCAGATTGTCGAC-(95-bp)-GCTAGCTACTGTTTGCCCCCGGGATGCAT; Early-1 plus PHA-4 (low), GCATGCTCGAGAGACGCAGATTGTCGAC-(95-bp)-GCTAGCTACTATTTGTCCCCGGGATGCAT; Late-2 plus PHA-4 (high), GCATGCTCGAGCTCTTTTTCCCATCGAC-(95-bp)-GCTAGCTACTGTTTGCCCCCGGGATGCAT; and Late-2 plus PHA-4 (low), GCATGCTCGAGCTCTTTTTCCCATCGAC-(95-bp)-GCTAGCTACTATTTGTCCCCGGGATGCAT.

Sequences chosen were the best match to the position weight matrix (PWM) generated by Improbizer, except Late-1 (which is based on the functional element in R07B1.9), P-1 (which is based on two overlapping PWMs), and the PHA-4 sites (which are based on a functional site in the *Ce-pax-1* promoter; J. Stevenson and S. E. M., unpublished data). PWMs from Improbizer are listed in Dataset S1. Complete details of all oligonucleotides and plasmids are available upon request.

#### Construction of transgenic lines

Several groups have reported artificial pharyngeal expression resulting from sequences present in the vector backbone of reporter constructs (e.g., Hope 1991). To minimize this effect, we routinely removed all vector sequence from our reporter constructs prior to injection into C. elegans. For our transcriptional fusions, we used a gene-specific oligonucleotide together with an *unc-54* 3′ oligonucleotide that anneals downstream of the *unc-54* 3′ cassette present in our plasmids (Fire et al. 1990) to PCR-amplify linear fragments for injection. For our enhancer constructs, we used an oligonucleotide that anneals approximately 200 bp upstream of the MCS of pAP.10 (Gaudet and Mango 2002) together with *unc-54* 3′ to amplify transgenes. We then digested the PCR products with either StuI or SphI to remove the remaining approximately 200 bp of vector sequence and gel-purified the desired fragment for microinjection. These adjustments ensured that there was no spurious expression in pharyngeal cells from Δ*pes-10::GFP* in the absence of an enhancer or when three copies of a random sequence (corresponding to the degenerate sequence CWNCAYKGA) were placed in front of the Δ*pes-10* promoter (Figure 4; data not shown).

Linear transcriptional reporters were injected at 0.5–1.0 ng/μl together with 30 ng/μl pRF4 (Mello et al. 1991) cut with EcoRI and 70 ng/μl sheared herring sperm DNA (Kelly et al. 1997). In all cases where expression of transgenes was compared, injections were performed under the same conditions. To establish transgenic lines, we picked Roller animals from the F2 generation. For all transgenes, a minimum of two independent lines were analyzed.

#### Estimating percent GFP expression

The transgenic marker that we used, *rol-6(su1006),* which confers a Roller phenotype (Kramer et al. 1990), does not allow us to identify transgenic embryos. Therefore, we estimated the fraction of transgenic embryos that express GFP as follows. Embryos from transgenic adults were collected and split into two samples. The first sample was scored for stage and GFP expression, while the second sample was allowed to develop and eventually scored for percent Roller animals. The percent Roller score was used as an estimate of the percent of animals that were transgenics. The percent transgenics with GFP expression was therefore estimated to be equal to (number of embryos expressing GFP) / ((total number of embryos scored) × (percent Roller)). Where reported in the text, numbers of transgenic embryos scored were estimated by this same approach.

## Supporting Information

Dataset S1 Position Weight Matrices from Improbizer (14 KB PDF).

Table S1 The 339 Microarray Positives (57 KB PDF).

Table S2 List of 37 Ph-E Genes and 34 Ph-L Genes (14 KB PDF).

Table S3 Conservation of Non-Coding Sequences in Ph-E and Ph-L Genes (22 KB PDF).

Table S4 Occurrence of Conserved Temporal Elements and Predicted PHA-4 Sites in Ph-E and Ph-L Promoter Regions (18 KB PDF).

Table S5 List of Genes Containing Conserved Sites of Different Motifs Within 500 bp Upstream of Their Predicted Start Codons (30 KB PDF).

### Accession Numbers

The LocusLink (http://www.ncbi.nlm.nih.gov/LocusLink) accession numbers for the genes and gene products discussed in this paper are *act-1* (LocusID 179535), C49G7.4 (LocusID 178809), *ceh-22*/Nkx 2–5 (LocusID 179485), *Ce-pax-1* promoter (LocusID 187105), *D-pax2* (LocusID 43825), *end-1* (LocusID 179893), F30H5.3 (LocusID 175207), F47D12.7 (LocusID 175891), K07C11.4 (LocusID 179198), LAG-1 (LocusID 177373), M05B5.2 (LocusID 187451), *myo-2* (LocusID 181404), *ncr-1* (LocusID 180719), *par-1* (LocusID 179912), *pha-4* (LocusID 180357), R07B1.9 (LocusID 181201), *rol-6(su1006)* (LocusID 174397), *skn-1* (LocusID 177343), T05E11.3 (LocusID 178014), *vestigial* (LocusID 36421), and *Drosophila* Su(H) (LocusID 34881).

## Abbreviations

*C*: cluster score threshold (Cluster-Buster setting)
Fox: forkhead box
*g*: gap parameter (Cluster-Buster setting)
GFP: green fluorescent protein
*m*: motif score threshold (Cluster-Buster setting)
Ph-E: set of early-expressed pharyngeal genes
Ph-L: set of late-expressed pharyngeal genes
PWM: position weight matrix
