# Supplementary material for: Whole-Genome Analysis of Temporal Gene Expression during Foregut Development
Source: PLoS Biol. 2004 Oct 19;2(11):e352. doi: 10.1371/journal.pbio.0020352 (PMC523228; doi:10.1371/journal.pbio.0020352)
Supplement: Dataset S1 — (14 KB PDF). [file pbio.0020352.sd001.pdf]

Supplemental Information

Position Weight Matrices from Improbizer

PHA-4 (from Ph-E and Ph-L, respectively):

7.4786 TGTTTGCC

a	0.003	0.003	0.003	0.040	0.003	0.003	0.113	0.259
c	0.003	0.003	0.003	0.113	0.040	0.003	0.442	0.296
g	0.003	0.990	0.003	0.186	0.003	0.990	0.296	0.223
t	0.990	0.003	0.990	0.662	0.954	0.003	0.149	0.223

8.1637 TTGTTTGC

a	0.127	0.003	0.003	0.003	0.003	0.003	0.003	0.065
c	0.250	0.003	0.003	0.003	0.003	0.003	0.003	0.682
g	0.127	0.065	0.990	0.003	0.065	0.127	0.990	0.003
t	0.497	0.928	0.003	0.990	0.928	0.867	0.003	0.250

Early-1:

7.4694 AGAGACGAAG

a	0.435	0.034	0.867	0.003	0.867	0.127	0.003	0.650	0.466	0.249
c	0.096	0.065	0.065	0.065	0.126	0.651	0.003	0.251	0.219	0.157
g	0.281	0.898	0.003	0.929	0.003	0.096	0.990	0.065	0.065	0.343
t	0.188	0.003	0.065	0.003	0.003	0.126	0.003	0.034	0.250	0.250

Early-2:

7.4500 TCTGACCGT

a	0.219	0.003	0.003	0.003	0.960	0.003	0.003	0.034	0.158
c	0.065	0.712	0.158	0.281	0.003	0.959	0.743	0.065	0.003
g	0.126	0.003	0.003	0.713	0.003	0.003	0.250	0.528	0.219
t	0.590	0.281	0.836	0.003	0.034	0.034	0.003	0.373	0.620

Early-3:

7.6078 AAAAAAGTGGGA

a	0.497	0.435	0.528	0.466	0.620	0.713	0.188	0.342	0.003	0.219	0.003	0.497
c	0.096	0.158	0.065	0.281	0.065	0.003	0.003	0.003	0.003	0.003	0.003	0.219
g	0.281	0.342	0.312	0.188	0.188	0.219	0.805	0.003	0.990	0.774	0.990	0.188
t	0.126	0.065	0.096	0.065	0.127	0.065	0.003	0.651	0.003	0.003	0.003	0.096

Late-1:

8.6489 GGGAGGCGGGGA

a	0.378	0.207	0.310	0.582	0.037	0.003	0.037	0.003	0.276	0.139	0.310	0.378
c	0.105	0.037	0.275	0.071	0.071	0.412	0.684	0.003	0.003	0.105	0.037	0.207
g	0.412	0.752	0.310	0.344	0.820	0.582	0.003	0.990	0.718	0.650	0.582	0.310
t	0.105	0.003	0.105	0.003	0.071	0.003	0.275	0.003	0.003	0.105	0.071	0.105

Late-2:

7.61 TTTTTCC

a	0.352	0.003	0.003	0.003	0.032	0.003	0.003
c	0.090	0.032	0.090	0.003	0.003	0.990	0.990
g	0.003	0.003	0.003	0.003	0.119	0.003	0.003
t	0.555	0.961	0.904	0.990	0.845	0.003	0.003

Late-3:

7.6973 TCTCGGAA

a	0.188	0.003	0.065	0.003	0.003	0.003	0.682	0.497
c	0.003	0.867	0.065	0.805	0.003	0.003	0.188	0.003
g	0.127	0.127	0.003	0.188	0.990	0.990	0.003	0.250
t	0.682	0.003	0.867	0.003	0.003	0.003	0.127	0.250

P1:

7.1263 TTTTCACCAG

a	0.185	0.211	0.029	0.003	0.003	0.912	0.029	0.003	0.315	0.341
c	0.133	0.029	0.237	0.029	0.757	0.081	0.964	0.964	0.237	0.159
g	0.211	0.341	0.003	0.029	0.185	0.003	0.003	0.029	0.185	0.393
t	0.471	0.419	0.730	0.938	0.055	0.003	0.003	0.003	0.263	0.107

7.1440 TGGGCTCAC

```
       a  0.250 0.144 0.109 0.003 0.003 0.215 0.038 0.743 0.039
       c  0.039 0.144 0.038 0.039 0.955 0.038 0.814 0.039 0.955
       g  0.321 0.392 0.462 0.920 0.039 0.003 0.003 0.145 0.003
       t  0.391 0.320 0.391 0.038 0.003 0.744 0.144 0.074 0.003
```

P2:

```
7.6779 TCTTCCAAA
       a  0.180 0.003 0.003 0.109 0.003 0.003 0.779 0.743 0.532
       c  0.003 0.497 0.039 0.003 0.955 0.990 0.179 0.003 0.180
       g  0.109 0.003 0.003 0.003 0.039 0.003 0.003 0.215 0.003
       t  0.708 0.497 0.955 0.885 0.003 0.003 0.039 0.039 0.285
```

The PWMs are shown with their associated Improbizer score and the 'best match' sequence to the PWM. The score is used as a measure of significance by comparing motif scores to scores generated by control runs of Improbizer against randomized sequence.
