# Supplementary material for: Whole-Genome Analysis of Temporal Gene Expression during Foregut Development
Source: PLoS Biol. 2004 Oct 19;2(11):e352. doi: 10.1371/journal.pbio.0020352 (PMC523228; doi:10.1371/journal.pbio.0020352)
Supplement: Table S1 — (57 KB PDF). [file pbio.0020352.st001.pdf]

Gene	Name	Predicted Product and/or Function	Expression (Ref)	LG
B0238.12		Trypsin inhibitor like	ND	V
B0507.1		EGF-like, Worm-specific repeat type 1	Pharynx (YK)	V
C01B10.5	*hil-7*	Histone H1.1	Ubiquitous (Jedrusik and Schulze, 2001)	IV
**C02B10.3**		EGF	ND	IV
**C02B8.4**	*hlh-8*	Helix-loop-helix protein involved in muscle development	Non-pharyngeal muscles and neurons (Harfe *et al.* 1998)	X
C02C2.3	*cup-4*	Neurotransmitter-gated ion-channel required for coelomocyte endocytosis	ND	III
C02D5.3		Glutathione S-transferase	ND	III
C03A3.1		Protein of unknown function	ND	X
C03A7.14	*abu-8*	DUF139 containing protein	ND	V
C03A7.4	*pqn-5*	DUF139 containing protein	ND	V
C03A7.7	*abu-6*	DUF139 containing protein	Pharynx (YK)	V
C03A7.8	*abu-7*	DUF139 containing protein	ND	V
**C03C10.5**		Protein of unknown function	ND	III
C04E6.12		Trypsin inhibitor like	ND	V
C04F12.9		RNase H	ND	I
C05E11.8	*flp-12*	FMRFamide-like peptide neurotransmitter	Non-pharyngeal neurons (Li *et al.* 1999)	X
C06E4.2		Protein of unknown function	ND	IV
C06G1.2		Protein of unknown function	ND	X
**C06G1.4**		Protein of unknown function	strong anterior expression, including pharyngeal cells (YK)	X
**C07D10.5**		Protein of unknown function	Intestine and weak pharynx (YK)	II
**C07E3.7**		Homeobox protein	ND	II
C10G8.2		Kunitz/Bovine pancreatic trypsin inhibitor	ND	V
C10G8.5	*ncx-2*	Na-Ca exchanger protein	Pharynx (YK)	V
**C10G8.6**	*ceh-34*	Homeodomain, similar to SIX2	Late Pharynx (Dozier *et al.*, 2001; C. Dozier, G. Cassata, G. Niklaus, H. Kagoshima, and T. Burglin, pers.	V

			Comm.)	
C14B9.2		Thioredoxin, disulphide isomerase	Pharynx (YK)	III
C14C11.8	*pqn-13*	DUF139 containing protein	Early pharynx muscle and marginal cells (YK, Ao *et al.*)	V
C14C6.5		ShTK containing protein	ND	V
C15H9.9		Protein of unknown function	Pharynx marginal cells and non-pharyngeal head cells (Ao *et al.*)	X
**C17E4.2**		Myosin heavy chain like	Broad embryonic (IH)	I
C18A11.1		Protein of unknown function	ND	X
**C18D1.1**	*die-1*	C2H2 zinc finger, required for morphogenesis of several tissues	Pharynx, hypodermis, muscle, intestine (Heid *et al.* 2001)	II
C18H9.6		Protein of unknown function	ND	II
C23H3.9		Metallo-beta-lactamase superfamily member	Pharynx (YK)	II
C24A3.2		Protein of unknown function	ND	X
**C24H12.1**		Cyclin-like F-box	ND	II
**C24H12.7**		Cyclin-like F-box	ND	II
C25A11.4	*ajm-1*	Apical junction proteins	Epithelia: pharynx, hypodermis, intestine (Mohler *et al.* 1998)	X
**C25B8.4**		C-type lectin	Pharynx (YK)	X
**C25G6.2**	*tsp-9*	Tetraspanin integral membrane protein	Neuronal (YK)	X
C27A2.5		4Fe-4S ferrodoxin, alpha defensin	ND	II
C27C12.6		Doublesex-like	Pharynx (JG, unpub obs)	X
**C30E1.5**		Protein of unknown function	ND	X
C30G12.1		Protein of unknown function	ND	II
C32H11.5		Protein of unknown function	ND	IV
**C33D9.8**		Human microtubule-vesicle linker CLIP-170 like, contains tropomyosin domain	ND	IV
**C34F6.6**		Protein of unknown function	ND	X
**C36A4.10**		Protein of unknown function	ND	III
C36H8.2	*inx-6*	Innexin	Pharynx (Starich *et al.*, 2001 and Phelan and	IV

			Starich, 2001)	
C37H5.13		Acyl-CoA thioesterase	ND	V
**C39F7.2**		Contains C3HC4 RING finger (zinc finger), fibronectin type III domain, and B-box zinc finger	ND	V
**C40A11.2**		Lipase (class 3), BTB/POZ, and K+ channel tetramerisation domains	ND	II
**C40A11.6**		BTB/POZ and K+ channel tetramerisation domains	ND	II
C44H4.1		Leucine Rich Repeat protein	Pharynx (Gaudet and Mango, 2002)	X
C45B11.2		Epoxide hydrolase like	Maternal/early embryo (YK)	V
**C46H11.8**		ShTK containing protein	Pharynx gland cells (Ao *et al.*)	I
C46H11.9		ShTK containing protein	Pharynx (YK)	I
C49G7.4		ShTK containing protein	Pharynx gland cells (Ao *et al.*)	V
C50B8.4		Protein of unknown function	ND	V
C53B7.1	*rig-3*	Neuronal IGCAM, member of immunoglobulin superfamily	Pharyngeal and non-pharyngeal neurons (Aurelio *et al.*, 2002)	X
C53C11.3	*ptr-5*	Patched related	Non-pharyngeal neurons (YK)	X
C53C9.2		Calponin family member	Body muscle (YK)	X
C54G4.4		Sushi repeats, C-type lectin domain	Intestine (YK)	I
C55A6.6		Alcohol dehydrogenase	ND	V
CD4.9		Low-density lipoprotein receptor, class A	ND	V
D1009.5		Tctex-1 (dynein light chain) family member	ND	X
D1054.8		Alcohol/short chain dehydrogenase	ND	V
D1054.9		Protein of unknown function	Pharynx (YK)	V
D2024.4		Protein of unknown function	ND	IV
D2092.6		Protein of unknown function	ND	I
D2096.6		Protein of unknown function	Pharynx (Gaudet and Mango, 2002)	IV
DY3.5	*pqn-26*	Protein of unknown function	ND	I
**E01G4.5**		Serpin (serine protease inhibitor) and lipocalin domains	Gonad/germline and maternal (YK)	II

E01G6.1		Kunitz/Bovine pancreatic trypsin inhibitor	Pharynx (YK)	X
**F01E11.1**		UDP-glucurosyltransferase	Broad but pharynx-enriched (YK)	X
F07C4.11		ShTK containing protein	ND	V
F07H5.6		Protein of unknown function	ND	II
F08B12.1		Glucagon/GIP/secretin/VIP family member	Pharynx (YK)	X
F08B12.4		Protein of unknown function	Pharynx (YK)	X
F08B4.2	*cdh-5*	Cadherin	ND	IV
**F08C6.3**		Vps52/Sac2 family	Gonad/germline (YK)	X
**F08G12.10**	*inx-2*	Innexin	Ubiquitous in embryo, pharynx in larva (Starich *et al.*, 2001 and Phelan and Starich, 2001)	X
F08G2.5		Protein of unknown function	ND	II
F09F3.6		Transthyretin-like	ND	V
F09F7.8		Protein of unknown function	ND	III
F10B5.3		C2H2 zinc finger	ND	II
F10F2.9	*pqn-29*	Protein of unknown function	ND	III
F10G8.8		PH domain	Pharynx (YK)	I
F11D5.1		Protein of unknown function	ND	X
F11E6.8		Protein kinase	Pharynx (YK)	IV
**F11G11.2**	*gst-7*	Glutathione S-transferase	Pharynx (YK)	II
F12F3.1	*exp-2*	Potassium ion channel	Pharynx muscle, intestinal muscle, neurons (Davis *et al.*, 1999)	V
**F13H10.1**		Protein of unknown function	ND	IV
F13H8.4		Protein of unknown function	ND	II
F14B4.1		LDL receptor-related protein	Pharynx (YK)	I
F16B4.8	*cdc-25.2*	Phosphatase, rhodanese-like	Non-pharyngeal (YK)	V
**F17A9.4**		NADH oxidase family member	ND	V
**F17E9.9**	*his-34*	H2B histone	ND	IV
**F18E9.2**	*nlp-7*	Neuropeptide-like protein	Non-pharyngeal neurons (Nathoo *et al.*, 2001)	X
F19C6.4		Neprilysin (zinc metallopeptidase)	ND	X
F19G12.3		Protein of unknown function, contains PAZ domain	ND	X
F20B10.3		Protein of unknown function	ND	IV

F21D5.9		C2H2 zinc finger	Pharynx, intestine, and hypodermis (Ao *et al.*)	IV
F21D9.5		Protein of unknown function	ND	V
F21H11.3	*tbx-2*	T-box family member	Pharynx (YK)	III
F22A3.1		ETS and Pointed domains	Pharynx, hypodermis, vulva (YK)	X
**F22F1.1**	*hil-3*	Histone H1-like	Pharynx, and gonad/germline and maternal (YK)	X
**F22F4.2**	*inx-3*	Innexin	Ubiquitous in embryo, pharynx in larva (Starich *et al.,* 2001 and Phelan and Starich, 2001)	X
**F23A7.5**		Protein of unknown function	ND	X
F25G6.6	*nrs-2*	Glutamine/Asparigine amidotransferase	ND	V
F26A10.2		C2H2 zinc finger	Pharynx (YK)	X
F26D10.11		Protein of unknown function	ND	IV
F26E4.12		Glutathione peroxidase	ND	I
F26F12.4		Protein of unknown function	Pharynx (YK)	V
F26G1.6		Neprilysin (zinc metallopeptidase)	ND	II
**F28B1.3**		Protein of unknown function	ND	V
**F28D1.2**		Protein of unknown function	ND	IV
F29F11.5	*ceh-22*	NK-2 Homeodomain protein	Pharynx muscle (Okkema and Fire, 1994)	V
**F30H5.3**		Serine inhibitor	Pharynx (YK)	III
F35A5.3	*abu-10*	DUF139 containing protein	Pharynx muscle (Ao *et al.*)	X
F35D11.2	*pqn-35*	Protein of unknown function	Pharynx (IH)	II
F35E12.5		DUF141 containing protein	ND	V
F36D4.3	*hum-2*	Unconventional myosin heavy chain, class V	Pharynx (YK)	V
**F37B1.2**	*gst-12*	Glutathione S-transferase	ND	II
F38A6.1	*pha-4*	Forkhead box protein, class A	Pharynx, hindgut, intestine (Horner *et al.* 1998, Kalb *et al.* 1998)	V
F38B6.1		Protein of unknown function	ND	X
F38H4.3		Protein of unknown function	ND	IV

**F39E9.5**		Mariner transposase	ND	II
**F39E9.6**		Neprilysin (zinc metallopeptidase)	ND	II
F39E9.7		Double-stranded RNA binding domain	ND	II
F40E10.2	*sox-3*	SRY box family member	ND	X
F40E10.5		DUF148 containing protein	ND	X
F40F9.10		Met-10+ like protein	ND	V
**F40G9.5**		DUF216 containing protein	ND	III
**F40G9.6**		Protein of unknown function	ND	III
F40H3.4	*fkh-8*	Forkhead box protein	Non-pharyngeal neurons (Hope *et al.* 2003)	II
**F40H3.5**	*hst-3*	Heparan sulphotransferase	ND	II
F41G3.10		ShTK containing protein	ND	II
F41H10.8	*elo-6*	Polyunsaturated fatty acid elongase	Pharynx (YK)	IV
F42G2.3		Protein of unknown function	ND	II
F42G9.9	*ptl-1*	Tubulin-binding Tau protein	Non-pharyngeal neurons (Goedert *et al.*, 1996)	III
**F43G9.11**	*ces-1*	C2H2 zinc finger	ND	I
F43H9.2		Aminotransferase	ND	V
F44A2.5		Initiation factor eIF-4 gamma, middle domain	Pharynx (IH)	V
**F44E7.2**		Protein of unknown function	ND	V
**F45F2.2**	*his-39*	Histone 2B	ND	V
F45G2.2		Myosin head (motor domain)	Pharynx muscle (JG, unpub obs)	III
**F45H11.1**		C2H2 zinc finger protein	ND	I
**F46F3.1**	*ceh-27*	Homeobox domain	Anterior/head of embryo (Harfe and Fire via WormBase)	V
F47F6.2	*lin-42*	PAS and PAC domains, required for L4/adult switch	Hypodermis (Jeon *et al.* 1999)	II
**F48C5.1**		Immunoglobulin superfamily member	Pharynx (YK)	X
F48E3.8		EGF-containing protein	Pharynx (YK)	X
F49D11.8	*cpn-4*	Calponin	ND	I
F49E10.2		Retinal pigment epithelial membrane protein	Pharynx (YK)	X
F52B11.5		Protein of unknown function	ND	IV
F53A9.3		Protein of unknown function	ND	X
F53B3.3		Protein of unknown function	Pharynx (IH)	X
F53H4.5		SAND domain	Pharynx (IH)	X
F54E2.2		Contains whey acidic protein, core region	Pharynx (YK)	V
F54E2.3	*pqn-43*	Immunoglobulin-like	Pharynx (YK)	V

F54F3.1	*nid-1*	Nidogen	Body wall muscle, neurons, pharynx and intestine (Kang and Kramer, 2000)	V
**F54H12.3**		Contains integrase core domain	ND	III
**F56A12.1**	*ceh-35*	Homeobox protein, six/sine oculis class	ND	V
**F56D12.4**	*jip-1*	SH3 and Phosphotyrosine interaction domains	ND	II
**F56D2.3**		Protein of unknown function	Pharynx (YK)	III
**F56H9.2**		Protein of unknown function	ND	V
F57B1.6		Protein of unknown function	ND	V
F58G4.1		Myosin	Pharynx (YK)	V
**F59B10.3**		Cadherin transmembranous domain	ND	II
F59C12.1	*cdh-9*	Cadherin	Pharynx (R. Babbar and J. Pettit, pers comm.)	X
H30A04.1	*eat-20*	EGF-like, paralog of crumbs/crb-1	Pharynx muscle and non-pharyngeal neurons (Shibata *et al.*, 2000)	X
**K01C8.2**		Contains worm specific repeat type I	ND	II
K01D12.11		Glutathione S-transferase	ND	V
**K02E10.2**	*hid-1*	Member of High temperature-induced Dauer formation gene class, novel protein	Non-pharyngeal neurons and pharynx (IH)	X
K04G11.2		Protein of unknown function	ND	X
K04G11.6		Protein of unknown function	ND	X
**K04H4.2**		Chitin-binding motifs	Pharynx (YK)	III
K06A1.3		Protein of unknown function	Pharynx (YK)	II
**K07C11.1**	*pax-1*	Paired box transcription factor	Pharyngeal marginal cells (Stevenson *et al.*, in prep)	V
K07C11.4		Esterase	Pharynx muscle and marginal cells, intestine, hindgut, and proximal somatic gonad (this paper)	V
K07D4.6		Protein of unknown function	ND	II

K08A8.2	*sox-2*	SRY box family member	ND	X
K08D9.4		Esterase	ND	V
K08F4.11	*gst-3*	Glutathione S-transferase	ND	IV
K08F4.7	*gst-4*	Glutathione S-transferase	ND	IV
K08F8.2		Basic-leucine zipper transcription factor	Pharynx (YK)	II
**K09B11.3**		Protein of unknown function	ND	IV
**K09F6.9**		Contains M protein repeat	ND	II
K10D11.1		DUF141 containing protein	ND	IV
K10D3.4		Kunitz/Bovine pancreatic trypsin inhibitor	Pharynx (YK)	I
**K10H10.3**	*dhs-8*	Alcohol/short chain dehydrogenase	ND	II
K11C4.2		Hly-III related protein (integral membrane protein)	ND	V
**K11G9.4**	*egl-46*	C2H2 zinc finger protein	Non-pharyngeal neurons (Wu *et al.*, 2001)	V
K11H12.5		Protein of unknown function	ND	IV
M01D1.2		Contains MATH domain	ND	II
**M02A10.3**	*sli-1*	Cbl proto-oncogene homolog	Non-pharyngeal (YK)	X
M02G9.1		DUF139 containing protein; keratin like	ND	II
M03D4.4		C2H2 zinc finger protein	Pharynx muscle (Ao *et al.*)	IV
M05B5.2		Protein of unknown function	Pharynx (Gaudet and Mango, 2002)	I
**M153.3**		ShTK containing protein	ND	X
M162.2		C-type lectin	ND	V
M195.2		C-type lectin	ND	II
M88.4		Contains phosphotyrosine interaction domain	Pharynx (YK)	III
R02F11.1		Protein of unknown function	Pharynx (YK)	V
R03C1.1		Protein of unknown function	ND	II
R03D7.6	*gst-5*	Glutathione S-transferase	ND	II
R04E5.7		Protein of unknown function	Body muscle or hyp? (YK)	X
R06F6.9		Acyl CoA binding protein, Enoyl-CoA hydratase/isomerase	Pharynx (YK)	II
R07B1.10	*lec-8*	Galectin	Pharynx (YK)	X
R07B1.9		Protein of unknown function	Pharynx muscle (this paper; Ao *et al.*)	X
R07C3.5		Protein of unknown function	ND	II
R07E3.2		Protein of unknown function	ND	X

R07H5.4		Protein of unknown function	ND	IV
R08F11.3		Cytochrome P450	ND	V
R09B5.5	*pqn-54*	DUF139 containing protein	ND	V
R09E10.5		AMOP and nidogen extracellular region domains	ND	IV
R09F10.2	*abu-9*	DUF139 containing protein	ND	X
R09F10.7	*pqn-57*	DUF139 containing protein	ND	X
R102.2		Protein of unknown function	ND	IV
R107.7	*gst-1*	Glutathione S-transferase	ND	III
R11G1.6		Protein kinase C, phorbol ester/diacylglycerol binding domain	Pharynx (YK)	X
R11G11.7	*pqn-60*	DUF148 containing protein	ND	V
**R12A1.3**		Kunitz/Bovine pancreatic trypsin inhibitor	ND	V
**R12H7.3**	*skr-19*	Skp1 homolog	Pharynx (YK)	X
**T01B11.2**		Aminotransferase, class III	ND	IV
T01B7.8		4Fe-4S ferrodoxin, alpha defensin	ND	II
T01D1.6	*abu-11*	DUF139 containing protein	ND	II
T03D8.4	*grl-14*	Ground-like, DUF398 containing	ND	V
T03F1.11		Protein of unknown function	Pharynx (YK)	I
T03G11.8	*zig-6*	Immunoglobulin-like	Body muscle (Aurelio *et al.*, 2002)	X
**T04B8.2**		Cyclin-like F-box	ND	II
T04C9.4	*mlp-1*	LIM domain	Pharynx (YK)	III
T04C9.6	*frm-2*	FERM domain (protein 4.1 family)	Pharynx (YK)	III
T04H1.6	*lrx-1*	Low-density lipoprotein receptor domain, class A	ND	V
**T05A6.1**	*cki-1*	Cyclin-dependent kinase inhibitor	Neurons, hypodermis, pharynx (Hong *et al.*, 1998)	II
**T05A7.4**	*hmg-11*	HMG-I/HMG-Y DNA-binding domain (A+T hook)	Vulva and broad early embryo (YK)	II
T05B4.11		ShTK containing protein	Pharynx (YK)	V
T05B4.12		ShTK containing protein	ND	V
T05B4.13		ShTK containing protein	ND	V
T05B4.3		ShTK containing protein	Pharynx (YK)	V
T05B4.8		ShTK containing protein	ND	V
T05C12.4		Protein of unknown function	ND	II
T05E11.3		Endoplasmin precursor (GRP94); Hsp90 protein	Pharynx, hindgut, intestine (Gaudet and Mango, 2002; YK)	IV

T06D8.3		PAP2-related phosphatase/phosphoesterase	Pharynx (YK)	II
T06E4.11	*pqn-63*	Protein of unknown function	ND	V
T06E4.7		Nematode 7TM chemoreceptor	Pharynx (YK)	V
T06E4.8		Protein of unknown function	Pharynx (YK)	V
T06E4.9		Protein of unknown function	ND	V
T08B1.2	*tnt-4*	Troponin T	(Bucher lab via WormBase)	V
T08E11.7		Cyclin-like F-box	ND	II
**T08G3.10**	*srw-35*	Serpentine receptor, class W	ND	V
**T09B4.5**		Protein of unknown function	Broad but pharynx-enriched (YK)	I
**T09B4.6**		Ser/Thr protein kinase	ND	I
T10E10.4		Chitin-binding domain	Broad but pharynx-enriched (YK)	X
T11B7.4	*eat-1*	LIM domain, homolog of ALP/Enigma	Pharynx muscle and body muscle (McKeown and Beckerle, pers comm)	IV
T11F9.12		Protein of unknown function	Pharynx muscle (unpub obs)	V
T12B5.8		Cyclin-like F-box	ND	III
**T14E8.3**		7 transmembrane receptor	ND	X
T14F9.4	*peb-1*	FLYWCH zinc finger	Pharynx, hindgut and vulva (Thatcher *et al.*, 2001)	X
T14G10.4		Transthyretin-like	ND	IV
**T14G12.4**	*fkh-2*	Fork head box, sloppy-paired homolog	AB and D descendants (Hope *et al.*, 2003)	X
T15H9.3	*hlh-6*	Helix-loop-helix DNA-binding domain	ND	II
**T16A1.9**		RNA-directed DNA polymerase	ND	II
T16G1.1	*pqn-67*	Contains worm-specific repeat type 1	ND	V
T17H7.4d	*gei-16*	Mpv17/PMP22 domain, involved in morphogenesis	ND	III
T18D3.4	*myo-2*	Myosin heavy chain, structural class	Pharynx muscle (Okkema *et al.*, 1993)	X
T20B3.2	*tni-3*	Troponin I	Body muscle (H. Kagawa, pers comm.)	V

T20G5.7		ShTK containing protein	Pharynx (YK)	III
**T22A3.8**	*lam-3*	Laminin related	Broad embryonic, but enriched in late embryo pharynx (YK)	I
T22B11.4		Protein of unknown function	ND	IV
**T22C8.3**		C2H2 zinc finger	ND	II
T23D8.8	*cfi-1*	ARID/BRIGHT DNA binding domain	Non-pharyngeal neurons, head cells, and pharynx muscle (Shaham and Bargmann, 2002)	I
T23F1.6	*pqn-71*	DUF139 containing protein	ND	V
T23G5.3		P21-Rho-binding domain	ND	III
**T24B8.6**	*hlh-3*	Helix-loop-helix domain	ND	II
T25E4.1		Protein of unknown function	Pharynx (YK)	II
**T25G12.8**		CCHC zinc finger	ND	X
T26C5.1	*gst-13*	Glutathione S-transferase	ND	II
**T27A1.2**		Protein of unknown function	ND	II
T27C5.10		Protein of unknown function	ND	V
T28B4.4		Claudin homolog, possible role in cohseion of apical junctions	ND	X
**T28B8.1**		Protein of unknown function	Pharynx (YK)	I
T28C12.6		Contains whey acidic protein, core region	ND	V
**VF13D12L.1**		myo-inositol-1-phosphate synthase	ND	II
W01C9.1		Protein of unknown function	ND	II
W01C9.3	*pqn-73*	Contains BRICHOS domain	Pharynx (YK)	II
**W03F8.10**		Protein of unknown function	ND	IV
W03F8.6		Protein of unknown function	Pharynx (IH)	IV
W04B5.1		Protein of unknown function	Intestine and weak pharynx (YK)	III
**W04H10.2**		Protein of unknown function	ND	II
W05E10.3	*ceh-32*	Homeodomain protein, related to Six3/6 subfamily	Anterior embryo (neurons, hypodermis, and pharynx; Dozier *et al.*, 2001; C. Dozier, G. Cassata, G. Niklaus, H. Kagoshima, and T. Burglin, pers. comm.)	V

W06D12.3	*fat-5*	Fatty acid desaturase	Pharynx (YK)	V
**W06F12.2**		Protein of unknown function	Pharynx (YK)	III
W06F12.3		Serine/threonine kinase	Pharynx (YK)	III
**W08E12.3**		2Fe-2S ferredoxin	Late embryonic pharynx (YK)	IV
**W08E12.5**		2Fe-2S ferredoxin	ND	IV
W10D9.1		Protein of unknown function	ND	I
**Y106G6H.13**		Protein of unknown function	ND	I
**Y37E11AR.6**	*vab-2*	Ephrin ligand	Non-pharyngeal neurons, pharynx (Chin Sang *et al.*, 1999)	IV
Y38H6C.14		Protein of unknown function	ND	V
**Y39G8C.4**	*col-87*	Cuticular collagen	ND	II
**Y40B1B.3**		Cyclin-like F-box	ND	I
**Y45G12C.2**	*gst-10*	Glutathione S-transferase	ND	V
**Y45G12C.3**		Glutathione S-transferase	ND	V
**Y46G5A.7**		Cyclin-like F-box	ND	II
**Y47D3A.1**		Protein of unknown function	ND	III
**Y47D3A.20**		Protein of unknown function	ND	III
**Y48E1B.11**		Protein of unknown function	ND	II
**Y49F6C.2**		Protein of unknown function	ND	II
**Y49F6C.3**		MATH and BTB/POZ domains	ND	II
**Y50E8A.5**		Gonadotropin, beta chain domain	ND	V
Y51A2D.11		Transthyretin-like	ND	V
**Y53F4B.7**		Protein of unknown function	ND	II
**Y54E2A.10**		Protein of unknown function	ND	II
**Y54E5A.1**		Fatty acid desaturase	ND	II
**Y66H1A.1**		Protein of unknown function	ND	IV
Y73F4A.1		DOMON domain (possible role in extracellular adhesion)	ND	IV
Y73F4A.2		DOMON domain (possible role in extracellular adhesion)	ND	IV
Y76A2B.2		Leucine rich repeat	ND	III
**Y9D1A.1**		Protein of unknown function	Pharynx (YK)	II
ZC101.1		Low density lipoprotein-receptor, class A	ND	II
ZC116.3		EGF-like and CUB domains; BMP-1 like	Neuronal? (YK)	V
**ZC132.6**		Peptidase family A16, CCHC zinc finger	ND	V
ZC250.1	*cyp-17*	Cyclophilin type peptidyl-prolyl cis-trans isomerase	ND	V
ZC443.2		Protein of unknown function	ND	V
ZC8.3		MYND type zinc finger	Broad but	X

			pharynx-enriched (YK)	
**ZC84.3**		Protein of unknown function	Non-pharyngeal neurons (IH)	III
ZK1025.7		DUF23 containing protein	ND	I
ZK1067.7	*pqn-95*	DUF139 containing protein	Pharynx muscle and marginal cells (Ao *et al.*)	II
ZK287.1		Protein of unknown function	Ubiquitous or very broad in embryos (YK)	V
ZK418.3		Protein of unknown function	ND	III
ZK662.2		Protein of unknown function	Pharynx (YK)	X
ZK673.7	*tnc-2*	Troponin C	Pharynx muscle (Terami and Kagawa, pers comm)	II
ZK682.5		Leucine rich repeat	Intestine (JG, unpub obs)	V
**ZK75.2**	*ins-2*	Insulin-related	Non-pharyngeal neurons, pharynx and vulva (Pierce *et al*, 2001)	II
**ZK792.7**		Ser/Thr protein phosphatase	ND	IV
**ZK816.3**		Protein of unknown function	ND	X
ZK816.4		Protein of unknown function	Pharynx (Gaudet and Mango, 2002)	X
ZK892.7		C2H2 zinc finger	ND	II
**ZK909.4**	*ces-2*	Basic leucine zipper (bZIP) transcription factor	Broad but pharynx-enriched (YK)	I

Supplemental Table 1. The 339 microarray positives. Genes in bold are new positives that are not part of the set from Gaudet and Mango, 2002. Genes in green are expressed in the pharynx, while those in red are non-pharyngeal. Descriptions of predicted products are summarized from entries in WormBase, version 110. Expression data is as described by others, by our GFP reporters, or from our interpretations of data from the Kohara lab Nematode Expression Pattern Database (NEXTDB).
