# Supplementary material for: Whole-Genome Analysis of Temporal Gene Expression during Foregut Development
Source: PLoS Biol. 2004 Oct 19;2(11):e352. doi: 10.1371/journal.pbio.0020352 (PMC523228; doi:10.1371/journal.pbio.0020352)
Supplement: Table S2 — (14 KB PDF). [file pbio.0020352.st002.pdf]

Gene	Gene Name	Protein Class
B0507.1		Other
C10G8.5	*ncx-2*	Channel/Receptor
C14B9.2		Enzyme
C14C11.8	*pqn-13*	DUF139
C44H4.1		Other
C46H11.9		ShTK
F08B12.1		Other
F10G8.8		Other
F11E6.8		Kinase/Phosphatase
F14B4.1		Other
F21H11.3	*tbx-2*	Trxn Factor
F26A10.2		Trxn Factor
F26F12.4		Unknown
F29F11.5	*ceh-22*	Trxn Factor
F30H5.3		Protease Inhibitor
F36D4.3	*hum-2*	Cytoskeletal/Muscle
F38A6.1	*pha-4*	Trxn Factor
F41H10.8	*elo-6*	Enzyme
F48E3.8		Other
F49E10.2		Other
F54E2.2		Other
K04H4.2		ECM
K07C11.1	*pax-1*	Trxn Factor
K07C11.4		Enzyme
K08F8.2		Trxn Factor
K10D3.4		Protease Inhibitor
M03D4.4		Trxn Factor
M05B5.2		Unknown
R02F11.1		Unknown
R07B1.10	*lec-8*	Other
T04C9.6	*frm-2*	Cytoskeletal/Muscle
T05E11.3		Other
T06D8.3		Kinase/Phosphatase
T20G5.7		ShTK
W01C9.3	*pqn-73*	Other
W06F12.2		Unknown
Y9D1A.1		Unknown

Supplemental Table 2A. List of 37 Ph-E genes. These ‘Early’ pharyngeal genes are all expressed at or before the formation of the pharynx primordium, in the bean to comma stage of embryogenesis. ‘Protein Class’ is as defined in Figure 2.

Gene	Gene Name	Protein Class
C03A7.7	*abu-6*	DUF139
C23H3.9		Enzyme
C49G7.4		ShTK
D1054.9		Unknown
E01G6.1		Protease Inhibitor
F08B12.4		Unknown
F12F3.1	*exp-2*	Channel/Receptor
F22A3.1		Trxn Factor
F44A2.5		Other
F45G2.2		Cytoskeletal/Muscle
F48C5.1		Other
F53B3.3		Unknown
F53H4.5		Trxn Factor
F54E2.3	*pqn-43*	Other
F58G4.1		Cytoskeletal/Muscle
H30A04.1	*eat-20*	Other
K06A1.3		ECM
M88.4		Other
R07B1.9		Unknown
R11G1.6		Kinase/Phosphatase
T03F1.11		Unknown
T04C9.4	*mlp-1*	Cytoskeletal/Muscle
T05B4.11		ShTK
T05B4.3		ShTK
T06E4.7		Channel/Receptor
T06E4.8		Unknown
T11B7.4	*eat-1*	Cytoskeletal/Muscle
T11F9.12		Unknown
T18D3.4	*myo-2*	Cytoskeletal/Muscle
T25E4.1		Unknown
W06D12.3	*fat-5*	Enzyme
ZK1067.7	*pqn-95*	DUF139
ZK662.2		Unknown
ZK673.7	*tnc-2*	Cytoskeletal/Muscle

Supplemental Table 2B. List of 34 Ph-L genes. These ‘Late’ pharyngeal genes are all expressed when the pharynx is almost completely developed or later, from the 3-fold stage of embryogenesis and on. ‘Protein Class’ is as defined in Figure 2.
