# Supplementary material for: Whole-Genome Analysis of Temporal Gene Expression during Foregut Development
Source: PLoS Biol. 2004 Oct 19;2(11):e352. doi: 10.1371/journal.pbio.0020352 (PMC523228; doi:10.1371/journal.pbio.0020352)
Supplement: Table S3 — (22 KB PDF). [file pbio.0020352.st003.pdf]

Table 3A.

GENE	501-1000	1-500
B0507.1	ND*	ND*
C10G8.5A	✓	✓
C14B9.2	ND*	✓
C14C11.8	✓	✓
C44H4.1	-	✓
C46H11.9	-	-
F08B12.1	-	✓
F10G8.8	-	✓
F11E6.8	-	✓
F14B4.1	-	-
F21H11.3	-	✓
F26A10.2	✓	✓
F26F12.4	✓	✓
F29F11.5	✓	✓
F30H5.3	-	-
F36D4.3B	-	✓
F38A6.1	-	-
F41H10.8	-	-
F48E3.8A	-	-
F49E10.2	✓	✓
F54E2.2	ND*	ND*
K04H4.2	✓	✓
K07C11.1	-	-
K07C11.4	-	✓
K08F8.2	✓	-
K10D3.4	NA*	NA*
M03D4.4	-	✓
M05B5.2	-	✓
R02F11.1	-	✓
R07B1.10	-	✓
T04C9.6	-	✓
T05E11.3	ND*	ND*
T06D8.3	-	✓
T20G5.7	-	-
W01C9.3	-	✓
W06F12.2	-	-
Y9D1A.1	NA*	NA*
Total:	8/31 = 26%	23/32 = 72%

Table 3B.

GENE	501-1000	1-500
C03A7.7	-	-
C23H3.9a	-	✓
C49G7.4	-	-
D1054.9A	-	✓
E01G6.1	✓	✓
F08B12.4	-	✓
F12F3.1	✓	✓
F22A3.1	-	✓
F44A2.5A	-	-
F45G2.2	-	-
F48C5.1	-	-
F53B3.3	-	✓
F53H4.5	✓	-
F54E2.3A	-	-
F58G4.1	-	-
H30A04.1	✓	✓
K06A1.3	-	✓
M88.4	-	✓
R07B1.9	-	✓
R11G1.4A	-	✓
T03F1.11	-	✓
T04C9.4A	-	-
T05B4.11	-	-
T05B4.3	-	-
T06E4.7	-	-
T06E4.8	-	✓
T11B7.4	-	-
T11F9.12	-	✓
T18D3.4	ND*	ND*
T25E4.1	-	✓
W06D12.3	-	✓
ZK1067.7	NA*	NA*
ZK662.2	✓	✓
ZK673.7	-	✓
Total:	5/32 = 16%	19/32 = 59%

Supplemental Table 3. Conservation of non-coding sequences in (A) Ph-E and (B) Ph-L genes. For all entries, a checkmark indicates the presence of 75% identity across at least 50 continuous base pairs, assayed using the VISTA alignment and plot program. ‘501-1000’ and '1-500' are the regions 501 to 1000 bp and 1 to 500 bp upstream of the predicted ATG, respectively. *NA = for some genes, sequence of *C. briggsae* orthologs could not be found. *ND = genes with upstream

neighbors < 1kb away; in these cases conservation of sequence could represent sequences associated with the neighboring gene and were ignored for this analysis.
