# Supplementary material for: Whole-Genome Analysis of Temporal Gene Expression during Foregut Development
Source: PLoS Biol. 2004 Oct 19;2(11):e352. doi: 10.1371/journal.pbio.0020352 (PMC523228; doi:10.1371/journal.pbio.0020352)
Supplement: Table S4 — (18 KB PDF). [file pbio.0020352.st004.pdf]

Gene	Temporal Element	PHA-4 Sites
B0507.1	E1 (-131), E2 (-102)	TGTTGAC (high, -138)
C10G8.5c	-	TGTTTAC (high, -415)
C14B9.2	-	TGTTTGT (med, -191)
C14C11.8	E1var (-406), E2 (-340)	TATTTGC (unk, -310)
C44H4.1	-	TATTTAC, TGTTTAT (unk, -179 , low, -122)
C46H11.9	E1var (-73)	TGTTTGT (med, -137)
F08B12.1	E2 (-190, -285)	TGTTGAT (unk, -85)
F10G8.8	-	TATTGAC (unk, -57)
F11E6.8	E1 (-320), E2 (-304)	TATTTGC (unk, -84)
F14B4.1	no upstream alignment available	
F21H11.3	L2(-268)	TATTTGT (low, -432)
F26A10.2	E2 (-231)	TGTTGAT (unk, -319)
F26F12.4	E1 (-161), E2 (-133, -321), L1 (-135), L2 (-265)	TATTGAC (unk, -345)
F29F11.5	-	TATTTGT (low, -355)
F30H5.3	E1var (-476)	TGTTTAC (high, -346)
F36D4.3c	-	TGTTGAC (high, -309)
F38A6.1	E1 (-88, -163), L1 (-159)	TGTTGAT, TATTGGC (unk, -115, unk, -63)
F41H10.8	E1 (-234), E2 (-464), L1 (-78)	TGTTTGC (high, -160)
F48E3.8	E1 (-94, -369)	TGTTGGT, TGTTGGT, TGTTTAT (unk, -62, unk, -145, unk, -361)
F49E10.2a	E1var (-162, -408)	TGTTTGC (high, -433)
F54E2.2	E1var (-67, -102)	TGTTTGT (med, -83)
K04H4.2a	E1 (-137, -237, -271), L1 (-332)	None
K07C11.1	E1var (-87)	TGTTTGC (high, -120)
K07C11.4	E1 (-61, -73), E2 (-50, -60, -177)	TGTTTGC (high, -151)
K08F8.2	E1 (-258), E2 (-86, -458)	TATTTGT (low, -234)
K10D3.4	no upstream alignment available	
M03D4.4	E1 (-334, -408), L1 (-442)	TGTTTGC (high, -488)
M05B5.2	-	TGTTGAC (high, -243)
R02F11.1	E2 (-239)	TGTTTGT (med, -412)
R07B1.10	-	TATTGAT, TGTTGAT (unk, -441, unk, -468)
T04C9.6	-	TGTTGAT (unk, -88)
T05E11.3	E1var (-75, -338, -403)	TGTTTGC (high, -80)
T06D8.3	E1 (-354, -421)	TGTTTAC (high, -443)
T20G5.7	no upstream alignment available	
W01C9.3	L2 (-403)	None
W06F12.2a	E1 (-368)	TGTTTGT (med, -165)
Y9D1A.1	no ortholog found	

Supplemental Table 4A. Occurrence of conserved temporal elements and predicted PHA-4 sites in Ph-E promoter regions. For simplicity, positions and sequences of PHA-4 sites are only given for *C. elegans* promoters. Only conserved temporal elements are listed. PHA-4 sites are also conserved, though in some cases the sequence of the site is different in *C. briggsae*. Not all PHA-4 sites are shown; for the sake of clarity, we preferentially list sites with the highest relative affinity. When both multiple unknown and low affinity sites are found, we indicate these. Sites

were identified with ClusterBuster (Frith et al., 2003) using the following parameters: The cluster threshold (C) and gap parameters (g) for all these motifs were 0 and 35 respectively. The motif threshold (m) used for Early-1 (E1), Early-2 (E2) and E1var was 6 and for PHA-4 was 5.

Gene	Temporal Element	PHA-4 Sites
C03A7.7	E1var (-428), L1 (-75)	TGTTTGC (high, -170)
C23H3.9	L2 (-51, -418)	TATTTGT (low, -26)
C49G7.4	-	TATTGGT (unk, -236)
D1054.9	E1 (-95)	TGTTTAT, TGTTGAT (unk, -224, unk, -287)
E01G6.1	L1 (-190), L2 (-57, -185)	TATTTGT (low, -90)
F08B12.4	L1 (-390), L2 (-356)	TGTTTGT (med, -492)
F12F3.1	-	None
F22A3.1	-	TGTTTAC (high, -18)
F44A2.5	-	TATTTGC, TATTGAT (unk, -270, unk, -281)
F45G2.2	-	TGTTGAT (unk, -369)
F48C5.1	-	TGTTTGT (med, -473)
F53B3.3	L2 (-17, -72)	TGTTTGC (high, -169)
F53H4.5	E2 (-276)	TGTTTGC (high, -111)
F54E2.3	E1var (-164), L2 (-388)	TGTTTAC (high, -381)
F58G4.1	-	TGTTTGC (high, -217)
H30A04.1	-	None
K06A1.3	E1 (-190, -230), E2 (-119)	TGTTTGT (med, -82)
M88.4	-	None
R07B1.9	L1 (-41)	TGTTTGC (high, -222)
R11G1.4	L1 (-200, -326)	TATTGAT, TATTTGC (unk, -44, unk, -128)
T03F1.11	L1 (-193, -213, -220), L2 (-292)	TGTTTGT (med, -216)
T04C9.4	L1 (-132)	TGTTTGC (high, -256)
T05B4.11	L2 (-15, -477)	TATTGAT (unk, -298)
T05B4.3	L2 (-372)	None
T06E4.7	-	None
T06E4.8	L2 (-88)	none
T11B7.4	no upstream alignment available	
T11F9.12	E1 (-82, -108, -216), L1 (-107)	TGTTTGT (med, -184)
T18D3.4	L1 (-41), L2 (-19, -116)	TGTTTGC (high, -425)
T23F1.6	-	TGTTTGC (high, -139)
T25E4.1	E1 (-366), L1 (-93), L2 (-438)	TATTTGT (low, -47)
W06D12.3	-	TGTTTGC (high, -246)
ZK1067.7	E1var (-78), L2 (-215)	TATTTGT (low, -441)
ZK662.2	L1 (-46)	TGTTTGC (high, -97)
ZK673.7	-	None

Supplemental Table 4B. Occurrence of conserved temporal elements and predicted PHA-4 sites in Ph-L promoter regions. For simplicity, positions and sequences of PHA-4 sites are only given for *C. elegans* promoters. Only conserved temporal elements are listed. PHA-4 sites are also conserved, though in some cases the sequence of the site is different in *C. briggsae*. Not all PHA-4 sites are shown; for the sake of clarity, we preferentially list sites with the highest relative affinity. When both multiple unknown and low affinity sites are found, we indicate these. Sites were identified using ClusterBuster (Frith et al., 2003) using the following parameters. The cluster threshold (C) and gap parameters (g) for all these motifs are 0 and 35 respectively.
The motif threshold (m) used for Late-1 (L1) was 6 whereas for Late-2 (L2) it was 7. The motif threshold used for PHA-4 was 5.
