# Supplementary material for: Whole-Genome Analysis of Temporal Gene Expression during Foregut Development
Source: PLoS Biol. 2004 Oct 19;2(11):e352. doi: 10.1371/journal.pbio.0020352 (PMC523228; doi:10.1371/journal.pbio.0020352)
Supplement: Table S5 — (30 KB PDF). [file pbio.0020352.st005.pdf]

Gene	Name	Expression	Reference
B0222.8	*col-10*	hypodermis	Liu et al., 1995
B0507.1*		early pharynx	YK
C09D1.1	*unc-89*	early pharnygeal muscle	Benian et al., 1996.
C14F5.1		not visible	YK
C16C4.2		NA	
C23H3.4		early pharyngeal	YK
C31A11.7		NA	
C42C1.2		NA	
C54D1.4	*alh-10*	not visible	YK
D2045.8		NA	
F10E7.4		non-pha head and early broad	YK
F11C3.3	*unc-54*	muscle, intestinal and rectal muscle cells	Miller et al., 1983
F11E6.8*		early pharynx	YK
F13E6.4		NA	
F13H8.4*		NA	
F18A11.4		NA	
F26F12.4*		early pharynx	YK
F31F6.7		NA	
F39D8.1	*pqn-36*	larval pharynx	YK
F41H10.8*		early pharynx	
F48F7.2		broad expression in embryos, but enriched in pharynx	YK
F49A5.8	*srw-23*	NA	
F49H12.5		complex embryonic expression, including hypodermis and pharynx	YK
F52D1.3	*pqn-40*	intestine and non-pharyngeal head cells	YK
F58A6.6		NA	
H13N06.3	*gob-1*	early intestine and pharynx	J. Kormish and J. McGhee, pers. comm.
K07D8.1	*mup-4*	late embryonic pharynx and muscle	Hong et al., 2001
K07G5.1		Early embryonic head, including pharynx	YK
K08A2.4		NA	
R04B5.4	*nhr-12*	NA	
T07D1.4	*fox-1*	broad expression in early embryos, pharynx-enriched	YK
T10G3.1		NA	
T14G11.1		NA	
T19A5.3		NA	
T25B9.9		embryonic intestine and pharynx (weak)	YK
W04G5.7		NA	

Y40B1B.6	*spr-5*	adult gonad	YK
Y54E10A.9	*vbh-1*	gonad	YK
Y66D12A.7		NA	
ZK381.5		NA	

Supplemental Table 5A. List of genes containing conserved Early-1, Early-2 and PHA-4 sites within 500 bp upstream of their predicted start codons. Green genes are expressd in the pharynx, while red genes are not; * = genes identified as positives in our microarray experiments. 'NA' = no expression pattern information available. For references, 'YK' refers to the Kohara group's Nematode Expression Pattern Database (NEXTDB); interpretation of the available *in situ* hybridization patterns was performed by the authors of this paper. The ClusterBuster (Frith et al. 2003) parameters for E1+E2 combined were C=3.5, m=6 g=35. The PHA-4 parameters were C=1.9, m=6, g=35.

Gene	Name	Expression	Reference
B0222.8*	*col-10*	Hypodermis	Liu *et al.*, 1995.
B0495.2		gonad/germline	YK
B0511.12		gonad/germline	YK
C01C4.3		not visible	YK
C01G8.2	*cln-3.2*	NA	
C04C3.3		larval intestine and pharynx	YK
C04E12.2		NA	
C05C10.1		NA	
C05D9.5	*ife-4*	NA	
C09D1.1*	*phm-1*	early pharyngeal muscle	Benian *et al.*, 1996.
C11H1.3		NA	
C13B9.2		NA	
C13G3.1		NA	
C14C11.8	*pqn-13*	early pharyngeal muscle and marginal cells	YK, Ao *et al.*
C15H9.10		NA	
C17A2.6		NA	
C25A11.1		NA	
C28C12.4		NA	
C30F12.5		NA	
C31A11.7*		NA	
C33G8.11	*nhr-107*	NA	
C33G8.6	*nhr-42*	not visible	YK
C35D10.16	*arx-6*	not visible	YK
C38D4.6	*pal-1*	many early embryonic cells, including pharyngeal lineages	
C43F9.6		not visible	YK
C46F4.2		intestine and other non-pharyngeal cells	YK
C47D12.2		gonad/germline	YK
C48E7.6		Mid-late embryonic intestine and pharynx	YK
C50F4.7	*his-37*	Broad, possibly ubiquitous	YK
C55B6.2	*dnj-7*	ubiquitous	YK
D1054.13		NA	
F02E8.6	*ncr-1*	Larval pharynx and proximal gonad	YK
F07C3.8	*str-94*	NA	
F09C6.9	*nhr-116*	NA	
F11A10.7		Broad expression in embryos; also in adult gonad	YK
F11C3.3*	*unc-54*	Body muscle	Miller *et al*, 1983.
F13G3.9	*mif-3*	Early embryos: body muscle, vulval muscles and pharyngeal muscle	Marson *et al.*, 2001.

F13H8.4*		NA	
F14B4.1		Early pharynx	YK
F16H11.2		NA	
F20G2.3		NA	
F25H8.3	*gon-1*	Distal tip cells, body muscle	Blelloch and Kimble, 1999.
F31D4.2		NA	
F31E8.4		NA	
F35H10.3		NA	
F36H2.2		NA	
F37B12.1		NA	
F37C12.3		not visible	YK
F40H3.4	*fkh-8*	Neuronal	Hope *et al.*, 2003.
F41H10.8*		Early pharynx	YK
F45E1.6	*his-71*	Intestine or gonad	YK
F45F2.3	*his-5*	NA	
F46C5.1		Adult gonad/germline	YK
F47D12.7		Early pharynx and late larval somatic gonad	YK
F47G6.2		NA	
F49E2.1		Intestine	YK
F52A8.5		not visible	YK
F52E1.13		Early intestine and faint pharynx	YK
F54D5.11		Gonad/germline	YK
F55G1.7		NA	
F58B3.9		Early pharynx	YK
F58E10.1		Larval pharynx	YK
F58H1.5		not visible	YK
F59B8.2		Broad in very early embryo, but becomes intestinal and pharynx enriched by comma stage	YK
F59C12.2	*ser-1*	Pharyngeal muscle, body muscle, neurons, vulva	Tsalik *et al.*, 2003.
F59F4.2		gonad/germline	YK
K07C11.4		Early pharynx	YK, this paper
K07D8.1*	*mup-4*	Late embryonic pharynx and muscle	Hong *et al.*, 2001.
K07G5.1*		Early embryonic head, including pharynx	YK
K08A2.4		Intestine or gonad	YK
K08F11.5		weak gonad/germline	YK
R03H4.1		NA	
R04B5.4*	*nhr-12*	NA	
R07B1.4	*gst-36*	NA	
R07B7.3	*pqn-53*	broad; possibly ubiquitous	YK
R13F6.4		NA	

T01C3.5		NA	
T02E9.2	*grl-7*	non-pharyngeal (can't determine cell type)	YK
T02G5.7		broad expression in early embryos, later becoming intestine-specific	YK
T04F8.4		NA	
T05A6.1	*cki-1*	Early pharynx and other tissues later	Hong *et al.*, 1998.
T06E4.4	*col-147*	Hypodermis	YK
T09A5.4		NA	
T09B4.4		NA	
T10E10.2	*col-167*	NA	
T10E10.5	*col-169*	NA	
T14A8.1	*ric-3*	Early pharynx muscle, body muscle and neurons	Halevi *et al.*, 2002., YK
T14G11.1*		NA	
T16G1.1	*pqn-67*	NA	
T19A5.2	*gck-1*	not visible	YK
T19D2.1		larval rectum	YK
T22C1.1		not visible	YK
T22C1.7	*jph-1*	Body muscle, neurons and pharynx muscle	GuhaThakurta *et al.*, 2002.
T27B7.4	*nhr-115*	NA	
W01A8.2		not visible	YK
Y102A11A.7		NA	
Y10G11A.2		NA	
Y116A8C.36		NA	
Y22D7AR.13	*ser-4*	Neurons, including pharyngeal neurons	Tsalik *et al.*, 2003.
Y34B4A.8		NA	
Y37A1A.1		NA	
Y37A1C.1		Weak early embryonic pharynx and stronger larval pharynx, gonad	YK
Y39B6A.21		NA	
Y39G8B.1		Intestine	YK
Y41D4B.16		NA	
Y45F3A.2	*rab-30*	NA	
Y54E10A.9*	*vbh-1*	gonad/germline	YK
Y54G11A.10	*lin-7*	larval intestine	Hope lab
Y57A10A.19		NA	
Y67A6A.2	*nhr-62*	not visible	YK
Y71F9B.7	*plk-2*	Broad in very early embryos, also in gonad/germline	YK
Y71G10AL.1		NA	
Y75B8A.3		Broad in early embryos, becoming pharynx specific in late embryos and larvae	YK

ZC487.5	*grl-8*	NA	
ZK1010.2		NA	
ZK1010.8		not visible	YK
ZK430.7		NA	
ZK792.3	*inx-9*	sheath cells	Starich *et al.*, 2001.
ZK836.2		gonad/germline	YK
ZK858.4	*mel-26*	NA	

Supplemental Table 5B. List of genes containing conserved E1var, Early-2 and PHA-4 sites within 500 bp upstream of their predicted start codons. Green genes are expressd in the pharynx, while red genes are not; * = genes identified in searches for Early-1, Early-2 and PHA-4 sites (see Table 6A, above). 'NA' = no expression pattern information available. For references, 'YK' refers to the Kohara group's Nematode Expression Pattern Database (NEXTDB); interpretation of the available *in situ* hybridization patterns was performed by the authors of this paper. The ClusterBuster (Frith et al., 2003) parameters for E1var +E2 combined were C=1, m=5.5 g=35. The PHA-4 parameters were C=2, m=6, g=35.

Gene	Name	Expression	Reference
B0414.7	*mtk-1*	NA	
C06E1.3		NA	
C09D4.3		distal gonad	YK
C10H11.5		NA	
C13D9.7	*ncx-8*	NA	
C13G3.1		NA	
C14H10.1		embryonic, non-pharyngeal	YK
C24A1.2		mid-embryonic pharynx	YK
C25B8.7		NA	
C25E10.5		broad non-pharyngeal expression in embryo	YK
C28C12.4		NA	
C42C1.5		NA	
C43F9.6		larval and adult gonad	YK
C50F4.8		NA	
C52B9.11		NA	
F08B12.3	*slo-2*	larval neurons and pharynx	Yuan *et al.*, 2000.
F09E8.2	*msh-5*	not visible	YK
F09G2.5		NA	
F10D7.5		late embryonic pharynx	YK
F14B8.6		late embryonic pharynx and intestine	YK
F18E2.3	*scc-3*	larval and adult gonad	Pasierbek *et al.*, 2003.
F22E5.16	*sri-57*	NA	
F28H1.3	*ars-2*	late embryonic expression, possibly ubiquitous	YK
F33E11.1	*nhr-15*	NA	
F35G12.4		larval and adult gonad	YK
F36H1.4	*lin-3*	larval and adult pharynx, gonad and vulval cells	Hwang and Sternberg, 2003.
F36H2.2		NA	
F37B12.1		NA	
F40E10.3	*csq-1*	body wall muscle and mid-late embryonic pharynx expression	YK
F40H3.4	*fkh-8*	non-pharyngeal neurons	Hope *et al.*, 2003.
F43C9.2		NA	
F46B6.9		NA	
F49E8.5	*dif-1*	broad expression in early embryos followed by expression in hypodermis and intestine	Ahringer, 1995.
F52E4.6	*wrt-2*	seam cells	Aspock *et al.*, 1999.
F55F3.3		larval and adult gonad	YK
F58G6.7		NA	

K07A1.8		larval and adult gonad	YK
K09H9.6	*lpd-6*	NA	
K12G11.3		larval and adult pharynx and intestine	YK
LLC1.1	*tra-3*	NA	
R07B1.9		late embryonic pharynx	this paper
T03F1.6		anterior region of embryos, including pharyngeal cells	IH
T04C9.4	*mlp-1*	NA	
T05C3.2		larval neurons (non-pharyngeal)	IH
T21D12.12		NA	
T23E7.2		late embryonic and larval expression in body muscle and pharynx (weak)	YK
T27F7.1		expression along body of late larvae and adults (non-pharyngeal)	YK
T28D6.9	*pen-2*	broad non-pharyngeal expression	Francis *et al.*, 2002.
W02F12.2		NA	
W04A4.5		Intestine	YK
W10C6.1	*mat-2*	broad expression in early embryos, also in larval and adult gonad	YK
Y32F6A.5		NA	
Y47G7B.3	*sri-60*	NA	
Y60A3A.8		NA	
Y75B8A.3		larval and adult pharynx	YK
Y77E11A.15	*col-106*	expressed along body of adult	YK
Y92H12BL.1		NA	
ZK1290.2	*tph-1*	larval pharyngeal and non-pharyngeal neurons	Sze *et al.*, 2000.
ZK430.7		NA	
ZK6.7		NA	
ZK836.2		adult gonad	YK

Supplemental Table 5C. List of genes containing conserved Late-1, Late-2 and PHA-4 sites within 500 bp upstream of their predicted start codons. Green genes are expressd in the pharynx, while red genes are not. ‘NA’ = no expression pattern information available. For references, ‘IH’ refers to Ian Hope’s expression pattern database (via WormBase) and ‘YK’ refers to the Kohara group’s Nematode Expression Pattern Database (NEXTDB); interpretation of the available *in situ* hybridization patterns was performed by the authors of this paper. The ClusterBuster (Frith et al., 2003) parameters for L1 +L2 combined are C=2, m=6 g=35. The PHA-4 parameters are C=2.5, m=6, g=35.
